# Gene Regulatory Network Characterization of Gastric Cancer’s Histological Subtypes: Distinctive Biological and Clinically Relevant Master Regulators

**DOI:** 10.3390/cancers14194961

**Published:** 2022-10-10

**Authors:** Sabino Russi, Luigi Marano, Simona Laurino, Giovanni Calice, Dario Scala, Graziella Marino, Alessandro Sgambato, Pellegrino Mazzone, Ludovico Carbone, Giuliana Napolitano, Franco Roviello, Geppino Falco, Pietro Zoppoli

**Affiliations:** 1 IRCCS-CROB Centro di Riferimento Oncologico della Basilica, 85028 Rionero in Vulture, Italy; 2 Unit of General Surgery and Surgical Oncology, Department of Medicine, Surgery and Neurosciences, University of Siena, 53100 Siena, Italy; 3 Biogem, Istituto di Biologia e Genetica Molecolare, Via Camporeale, 83031 Ariano Irpino, Italy; 4 Department of Biology, University of Naples ‘Federico II’, 80126 Naples, Italy; 5 Department of Molecular Medicine and Health Biotechnolgy, Università di Napoli Federico II, 80131 Naples, Italy

**Keywords:** gastric cancer, molecular classification, prognostic biomarkers, master regulator, gene expression profile

## Abstract

**Simple Summary:**

The prognosis of advanced gastric cancer patients remains unfavorable. Molecular heterogeneity has proven to be a major determinant of clinical outcomes. We characterized the transcriptome of the two major subgroups by highlighting the different biological and molecular pathways. We explored their association with clinicopathological features and survival. This comparative study aimed to define a reproducible in silico analysis so that the molecular mechanisms underlying carcinogenesis, disease natural history and the identification of new therapeutic targets can be traced.

**Abstract:**

Gastric cancer (GC) molecular heterogeneity represents a major determinant for clinical outcomes, and although new molecular classifications have been introduced, they are not easy to translate from bench to bedside. We explored the data from GC public databases by performing differential gene expression analysis (DEGs) and gene network reconstruction to identify master regulators (MRs), as well as a gene set analysis (GSA) to reveal their biological features. Moreover, we evaluated the association of MRs with clinicopathological parameters. According to the GSA, the *Diffuse* group was characterized by an epithelial-mesenchymal transition (EMT) and inflammatory response, while the *Intestinal* group was associated with a cell cycle and drug resistance pathways. In particular, the regulons of *Diffuse* MRs, such as *Vgll3* and *Ciita,* overlapped with the EMT and interferon-gamma response, while the regulons *Top2a* and *Foxm1* were shared with the cell cycle pathways in the *Intestinal* group. We also found a strict association between MR activity and several clinicopathological features, such as survival. Our approach led to the identification of genes and pathways differentially regulated in the *Intestinal* and *Diffuse* GC histotypes, highlighting biologically interesting MRs and subnetworks associated with clinical features and prognosis, suggesting putative actionable candidates.

## 1. Introduction

Gastric cancer (GC) was responsible for 769,000 deaths in 2020, ranked sixth for incidence and third for mortality [1]. Although treatment approaches have improved, the prognosis of advanced GC patients remains unfavorable, with a 5-year survival rate of less than 5% [2]. GC is characterized by both intra- and inter-tumoral heterogeneity [3]; thus, several classification systems have aimed to categorize different subtypes from a morphological point of view. Among them, the Lauren classification is the most widely used, and it separates GCs into *Intestinal*, *Diffuse* and mixed subtypes [4]. Additionally, a classification from the World Health Organization (WHO) [5] has been recently updated with a more systematic approach to stratifying GC into five main histological subtypes: the *Intestinal* subtypes from the Lauren classification (tubular, papillary and mucinous) and Lauren’s *Diffuse* types (poorly cohesive, signet ring cells and mucinous). WHO classification also includes mixed carcinoma and other less frequent gastric tumors. Nonetheless, categorization into a few macro-groups is burdened by excessive internal heterogeneity, resulting in conflicting evidence based on histopathological phenotypes for predicting patient prognosis or response to therapy. Additionally, the GC diversity also hinders the identification of prognostic markers from a molecular point of view (e.g., p53 or E-cadherin). Next-generation sequencing contributes to the establishment of new molecular classifications of gastric cancer, focusing on biological characteristics. The Cancer Genome Atlas (TCGA) identified four genomic gastric cancer subtypes: Epstein-Barr virus-associated (EBV), microsatellite instable (MSI), genomically stable (GS) and chromosomal instability (CIN) groups [6]. On the other hand, the Asian Cancer Research Group (ACRG) focused on gene expression profiles and classified gastric cancer into four subtypes with distinct clinical outcomes: microsatellite stable (MSS), epithelial-mesenchymal transition (EMT), MSI, MSS/p53 active and MSS/p53 inactive. This approach opens new possibilities to treat the disease in a tailored way [7]. Although, in recent years, many research efforts have proven that distinct molecular groups showed peculiar clinicopathological as well as prognostic characteristics [8,9,10,11,12,13,14,15], the integrative analysis of multiple genomic and proteomic datasets still remains expensive and quite complicated to translate into the clinical setting [16,17]. In this view, since histological assessment remains a cheap and widespread method, it would be of immense interest to thoroughly research the molecular differences between the *Intestinal* and *Diffuse* subtypes and the underlying gene networks responsible for the different biological behaviors. To the best of our knowledge, few studies have focused on the characterization of molecular differences within Lauren’s classification [18,19], and a reconstruction of the signaling networks differentially active between subtypes is lacking. Therefore, we more deeply investigated the associations of Lauren’s histotypes with clinicopathological features, searching for differentially expressed gene lists accordingly weighted. Additionally, we traced the transcriptional subnetworks characterizing the *Intestinal* and *Diffuse* subtypes and explored their associations with clinicopathological features and survival. This comparative study aimed to define a reproducible analysis so that the molecular mechanisms underlying carcinogenesis, disease behavior and the identification of new therapeutic targets can be traced.

## 2. Materials and Methods

### 2.1. Data Collection

TGCA (https://www.cancer.gov/tcga (accessed on 15 June 2021)) and the Genotype-Tissue Expression (GTEx) project (https://www.gtexportal.org/home/ (accessed on 15 June 2021)) are repositories, collected with high throughput, of clinical data of many tumors and normal tissues from many organs. Although unifying cancer and normal RNA sequencing data from diverse sources represents a bioinformatics challenge, TCGA and GTEx RNA-seq data were successfully merged by Wang et al. enabling cross-study analysis of RNA-sequencing data [20]. From the UCSC Xena browser (https://xenabrowser.net/datapages/ (accessed on 15 June 2021)), we retrieved the dataset (TCGA TARGET GTEx transcript expression by RSEM using UCSC TOIL RNA-seq recompute), clinical data and gene annotations. After crossing the RNA-seq dataset and clinical data, we obtained the data of 380 gastric cancer RNA-seqs, 32 adjacent normal (non-diseased) tissues and 112 normal samples. All the information about TCGA samples’ clinical data, pathology reports and tissues were easily retrievable by the cBioPortal (https://www.cbioportal.org/ (accessed on 15 June 2021)) website, while the “GTEx Tissue Harvesting Work Instruction” provided all the available information about the GTEx tissues. From the GEO repository [21], we retrieved the ACRG (GSE62254) [7] and GSE15459 [22] datasets containing 300 and 200 GCs, respectively.

### 2.2. Patient Selection and Study Design

This study included GC patients who underwent upfront gastrectomy for a malignant disease nbetweem January 2014 and March 2019 and were collected in the TGCA dataset. The GSE62254 dataset included *n* = 300 primary independent GC specimens at the time of the total or subtotal gastrectomy at Samsung Medical Centre, Seoul, South Korea, from 2004–2007 (all the tissue specimens were in a chemo-naïve state during the primary resection of gastric cancer). Case selection criteria were histologically confirmed adenocarcinoma of the stomach, surgical resectioning of primary GC, age ≥18 years and complete pathological, surgical, treatment and follow-up data. Patients in the GSE15459 dataset from the National Cancer Centre in Singapore had a median follow-up period of 13.47 months, and 91 patients died by the end of the study period. Histopathological data are provided in Ooi CH et al. [22].

### 2.3. Data Processing

RNA-seq and clinical data of the GTEx and TCGA databases were used to investigate transcriptomic profiles of the GC and normal gastric tissue, as performed by Russi et al. [23]. The previous histological categorizations were:

- Signet-Ring Cells (SRC),
- Diffuse (poorly cohesive, not SRC)
- Intestinal mucinous,
- Intestinal papillary,
- Intestinal tubular,
- Intestinal not otherwise specified (iNOS),
- Stomach not otherwise specified (sNOS).

Initially, the sNOS and SRC samples were filtered out due to the missing histology and unclear different behaviors [24], respectively. From now on, we refer to the poorly cohesive without SRCs as “*Diffuse*” (*n* = 62). On the other hand, *Intestinal* samples behave in a heterogeneous way, considering the association with clinical features and the survival results. We filtered out the papillary samples due to their scarcity (*n* = 7). Moreover, by performing a differential expression analysis (*p*-value < 0.05 and |FC > 1.5|), we included, in the “*Intestinal*” category, the tubular samples, representing most of the *Intestinal* adenocarcinomas [25], together with the iNOS or mucinous samples. Interestingly, compared with the tubular samples, mucinous samples showed 2828 DEGs while iNOS showed only 511. This result, together with the differences in survival and clinicopathological features (Supplementary Materials Figures S1–S6), suggested the exclusion of the mucinous samples from the *Intestinal* subgroup and further investigation of the possibility of retaining the iNOS. In this way, after differential gene expression analysis (adjusted *p*-value < 0.05 and |FC| > 1.5) between the *Diffuse* and the union of the *Intestinal* (tubular and iNOS) samples, we created a Venn diagram (in Supplementary Materials Figure S7). The 6827 DEGs, resulting from the comparison between the *Diffuse* vs. *Intestinal* (merged), showed great overlap with the *Diffuse* vs. tubular (88%) and the *Diffuse* vs. iNOS genes (80%). From now on, we refer to the tubular and iNOS as “*Intestinal*” (*n* = 140). Our final categorization, based on molecularly homogeneous histological types, was:

- *Diffuse* (poorly cohesive, not SRC),
- *Intestinal* (tubular and iNOS).

The rationale of the study is summarized through a schematic workflow in Supplementary Materials Figure S8.

### 2.4. Statistical Analysis

#### 2.4.1. Association with Clinical Features and Survival Analyses

We evaluated, with chi-square test, the association of the selected clinical variables and the histological type. We depicted the significant association with correlation plots. Moreover, by multivariate Cox proportional hazards analysis, we calculated the overall survival (OS), the disease-specific survival (DSS), the disease-free survival (DFS) and the progression-free interval (PFI) associated with the above clinical features. We applied the Akaike information criterion (AIC) based on a stepwise procedure to obtain the best candidate for the final Cox proportional hazards models, which were depicted as forest plots.

#### 2.4.2. Differential Expression Analysis and Venn

The regression-like model implemented by the *edgeR* [26] was used to perform differential expression analysis on RNA-seq data, while the *limma* implementation was used for the microarray data. According to the clinical data, we selected 62, 135 and 75 *Diffuse* samples and 140, 146 and 99 *Intestinal* samples from the TCGA, ACRG and GSE15459 datasets to perform a differential expression analysis. According to the explorative analysis, the pathologic stage and N feature of the TNM staging system were used as blocking factors in the TCGA data generalized linear modeling. A gene was considered as differentially expressed (DEG) if (1) corrected (FDR) *p*-value < 0.05 and (2) expression change > |1.5|-fold (log2FC > |0.58|).

#### 2.4.3. Gene-Set Enrichment Analyses and Master Regulator Analyses (MRA)

The overrepresentation of the Molecular Signatures Database’s (MSigDB) [27] hallmark (HM), gene ontology (GO), KEGG pathways, motifs, miRNA targets, chromosome position and immune system gene sets for each DEG list was obtained by applying the *ClusterProfiler* [28]. We considered statistically significant the gene sets resulting from the analysis of the TCGA data with an FDR adjusted *p*-value < 0.05. We retrieved, from the *aracne.networks* [29], the AP-ARACNE [30] inferred networks for the TCGA-STAD database. There are 6054 inferred subnetworks called regulons, which have HUB transcription regulators (TFs, co-TFs, etc.) called master regulators (MRs). Exploiting the *mra* function implemented in the *corto* [31], we scored the enrichment of each candidate MR in subgroups and samples according to the *logcpm* (logarithmic counts per million) of the genes in the dataset. The enrichment score (ES) reflected the degree to which a regulon was overrepresented at the top or bottom of a ranked list of genes. The higher the ES, the more active the subnetwork associated with the candidate MR. Moreover, to highlight the biological function of the MR’s regulon, we evaluated its overlap with the enriched gene sets. A hypergeometric test was performed to identify the significant overlaps. Statistical analysis was performed using the computing environment R (R Core Team, Vienna, Austria) [32].

#### 2.4.4. Single Sample Gene Set Enrichment Analysis (ssGSEA) and Single Sample Master Regulator Analysis (ssMRA)

To better describe the heterogeneity of the two groups, we performed both ssGSEA and ssMRA to obtain the activation or deactivation normalized enriched score (NES) for each gene set or each MR’s inferred small subnetwork (regulon) in each sample.

#### 2.4.5. Association of MRs with Clinical and Survival Features

We evaluated, by linear modeling, the association of the selected clinical variables and each MR’s profile obtained by ssMRA. We depicted selected interesting associations with a *ggplot2* [33]. Moreover, we described by univariate Cox analysis the overall, disease-specific, disease-free and progression-free survival associated with the MRs’ NES. Selected results are depicted as forest plots.

## 3. Results

### 3.1. In Silico-Refined Histological Subtypes Have Similar Clinicopathological Characteristics

A multivariate analysis was performed to evaluate the association of several clinicopathological features with the two histological subgroups. A total of 380 GC patients were eligible for our study: 62 were categorized as *Diffuse*, 12 as signet, 139 as stomach NOS, 17 as *Intestinal* mucinous, 7 as *Intestinal* papillary, 71 as *Intestinal* tubular, 69 as *Intestinal* NOS and 3 with no clinicopathological annotation. The patient characteristics and clinicopathological data are in Supplementary Materials Table S1. The man-to-woman ratio was 2/1. Around 60% were proximal while 40% were distal; 43% of tumors were the *Intestinal* type, and >50% were pathological stage ≥III. An association of the histological groups described above resulted in the pathologic stage, lymph nodes ratio (Figure S1), and with a primary therapy outcome (χ^2^ *p*-value < 0.01). Via multivariate Cox proportional hazards analysis, we investigated the association of clinical features with the overall survival (OS), disease-specific survival (DSS), disease-free survival (DFS) and progression-free interval (PFI). The best-fit model according to Akaike Information Criterion (AIC) for the overall survival comprises the histology and pathologic stage (global *p*-value = 1.3 × 10^−06^), while histology, pathologic stage and microsatellite status best model the DSS (global *p*-value = 1.4 × 10^−06^). Forest plots of the stepwise selected models are depicted in Supplementary Materials Figures S2–S5. According to the data processing paragraph, a total of 202 patients were clearly identified: 62 were categorized as *Diffuse* and 140 as *Intestinal* (including 71 tubular histotypes). The association profiles of the new *Intestinal* (Figure S6) and old *Intestinal* tubular and iNOS (Supplementary Materials Figure S1) with lymph nodes ratio and pathologic stage were clearly overlapping; thus, supporting our choice of merging the latter. The patients’ characteristics and clinical data for each new histological group are represented in Table 1.

Almost 70% of tumors were the *Intestinal* type. The man-to-woman ratio was 2:1 in the *Intestinal* and about 1:1 in the *Diffuse* GC. About 60% of the *Intestinal* GC were proximal, whereas GC localization was quite similar between the two sites in the *Diffuse* GC. Pathological stage ≥ III represented over 60% for both histological types.

Overall, there was no significant association between the histological (*Diffuse* and *Intestinal*) types and clinical data according to the χ^2^ test as well as with survival. Therefore, no other variables influenced the downstream analyses which focused on the molecular and functional characterization of GC subtypes.

### 3.2. Functional Enrichment Highlighted a Different Biological Behavior for the Two Histological Subtypes

**Figure 1:**
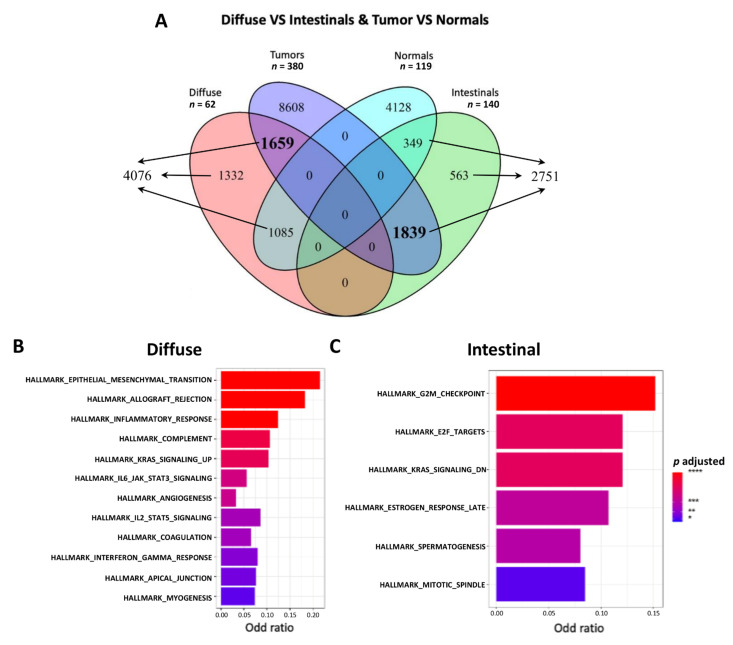
(**A**) Venn diagram showing the intersection among the genes differentially upregulated in *Diffuse* or *Intestinal* samples and in GC (tumor) samples from TGCA, STAD or normal ones from GTEx datasets, respectively. The number of genes in bold indicates distinctive *Diffuse* tumors and *Intestinal* tumors as compared with normal tissues. (**B**,**C**) Bar plot of hallmark categories enriched with upregulated genes in the *Diffuse* or *Intestinal* subtypes. N is the number of samples.

We performed several hierarchical steps of differential gene expression and functional enrichment analyses to evaluate the biological differences between the two GC histological subgroups and pinpoint respective candidate biomarkers. We first defined the differentially expressed genes between the GC (both *Diffuse* and *Intestinal* samples) and the normal mucosa samples (adjusted *p*-value <0.05 and |FC| > 1.5) by obtaining 12,106 upregulated and 5562 downregulated genes. We subsequently defined 4076 upregulated genes in the *Diffuse* vs*. Intestinal* samples and 2751 upregulated genes in the *Intestinal* vs. *Diffuse* samples. Finally, after the intersection between the GC upregulated and *Diffuse* or *Intestinal* upregulated genes, according to the Venn diagram in Figure 1A, we defined 1659 upregulated genes in both GC vs. normal mucosa and *Diffuse* vs. *Intestinal* samples as a *Diffuse* signature. 

Similarly, we defined as an *Intestinal* signature 1839 upregulated genes in both GC vs. healthy mucosa and in the *Intestinal* vs. *Diffuse* samples (Supplementary Materials File S1). Among the upregulated genes in GC vs. healthy mucosa (normal), around 25% are subtype-specific, defining two refined lists of GC histology-related candidate biomarkers. Interestingly, as suggested by the literature, ERBB2 results were strongly upregulated in *Intestinal* GC and CDH1 downregulated in the *Diffuse* type. To capture the relationships among the DEGs and gain biological insight into the global gene expression patterns that differentiate the *Diffuse* from *Intestinal* GC specimens, we performed a gene set analysis (GSA) using the mSigDB gene sets comprising, among others, hallmark and GO gene sets. The GSA results (adjusted *p*-value < 0.05) are reported in Table 2.

*Diffuse* tumors appeared to enrich a higher number of gene sets for all categories except for the positional one. Hallmark categories related to the epithelia-mesenchymal transition (EMT) and cellular cycle defined a clear biological difference between the two GC subtypes (Figure 1B,C). On the other hand, in the *Intestinal* tumors, we found enriched gene sets associated with cell cycle regulation, division and proliferation. Indeed, as recently reviewed, E2F’s transcription factor is a critical regulator of genes essential for cell cycle progression and control of cell proliferation [34]. Overall, evaluating the hallmarks and other ontologies, the *Diffuse* subtype seemed to be mainly characterized by an invasive phenotype, whereas the *Intestinal* subtype showed a proliferative one [35]. Moreover, the other gene sets (GO and pathways) depicted some remarkable differences between *Diffuse* and *Intestinal* GC, falling into the same biological features of the enriched hallmarks (Supplementary Materials Files S2 and S3). Among the top enriched pathways in the *Diffuse* type of GC, we found a signature related to multi-cancer invasiveness and extracellular matrix organization. Finally, we found some active pathways involved in immune system cell regulation as well as GOs relative to immune cell regulation and extracellular matrix organization (Supplementary Materials File S2). The pathways characterizing the *Intestinal* GC, in addition to other cancer-related pathways, were involved in cell cycle regulation and response to growth factors and hormones (EGFR, estradiol, progesterone). Indeed, the high endogenous estrogen exposure in women was indicated to be a protective factor, which explains the higher incidence of *Intestinal* GC in men [36]. Regarding GOs, peculiar for the *Intestinal* type of GC were those involved in cell division, chromosome segregation and embryo development, delineating a different biology for this GC histological type (Supplementary Materials File S3). Overall, based on DEGs and functional enrichment through GSA, we highlighted two distinct biological phenotypes. An invasive behavior is characterized by the upregulation of EMT-related genes in the *Diffuse* type, and a proliferative behavior is characterized by the upregulation of cell cycle-related genes in the *Intestinal* type.

### 3.3. Gene Regulatory Networks Highlights Different Putative Hub Genes

**Figure 2:**
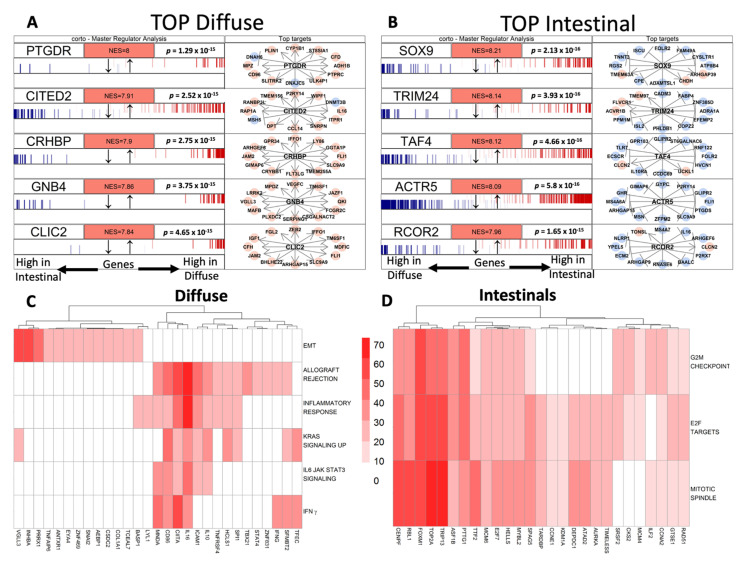
(**A**,**B**) *Intestinal* and *Diffuse* top master regulators. Each network is indicated by its MR. The genes in each network are shown in a barcode-like diagram showing all transcriptome genes from most downregulated (left) to most upregulated (right). Positively (red) and negatively (blue) correlated targets are overlaid on the differential expression signature as bars of different colors. Normalized enrichment score (NESes) and p-values are also indicated. To the right, the twelve highest-likelihood network putative targets of each MR are shown in red if upregulated or in blue if downregulated and with a pointed arrow if predicted to be activated by the centroid protein or with a blunt arrow if predicted to be repressed. (**C**,**D**) Heatmaps of the biological characterization of the top MRs in *Diffuse* and *Intestinal* subgroups. The intensity of the red color is proportional to the overlap between the genes in the regulon of the MR and the ones in the hallmark gene set.

We aimed to focus on MRs eligible for pathway-targeting therapy. First, we pictured the gene interdependence and exploited the inferred GC gene regulatory network. According to Carro M.S. and Chen J.C. [37,38], we obtained a set of putative MRs, each activating or repressing its inferred small subnetwork known as a regulon. In Figure 2A,B, as an example, we reported the top five MRs in *Diffuse* and *Intestinal* GCs. A complete MR list is reported in Supplementary Materials File S4.

Out of the 3608 significant MRs (*p*-value <0.01), 2058 were active in *Diffuse* GC while 1550 were active in *Intestinal* GC. Beyond the statistical significance of MRs, we focused our study on those that were likely relevant to cancer biology features by overlapping each MR’s regulon with significantly enriched gene sets from the GSA analysis. In this way, we aimed to give a biological characterization of the data-driven regulons which were significantly overrepresented by MR analysis. Hallmark gene sets, more than the others, immediately summarized and suggested specific well-defined biological states; thus, we focused on MRs overlapping with them (Figure 2C,D). Overlaps with the other gene set collections are reported in Supplementary Materials Figures S9–S18. Among those with the highest enrichment score, the regulon VGLL3, an unfavorable prognostic marker in GC [39], resulted in the highest association with EMT (Figure 2C) in *Diffuse* GCs as well as with the invasiveness signature resulting from the interactions between cancer cells and the microenvironment (Supplementary Materials Figure S10). We also found a significant overlap between the HLF’s targets and the VGLL3 regulon (Supplementary Materials Figure S11). In line with the hallmarks, VGLL3, INHBA and PRRX1, *Diffuse* GC regulons intersected with GOs relative to collagen synthesis and extracellular matrix organization. This biological phenotype seemed to be also regulated by miRNAs. Indeed, among the significantly enriched motifs overlapping with VGLL3, INHBA and PRRX1 regulons, we found MIR5682, MIR29A_3P and MIR29B_3P/MIR29C_3P ones (Supplementary Materials Figure S11). These miRNAs shared 174 target genes, which were mainly involved in extracellular matrix organization. Three MRs, namely CD86, CIITA and IL-16, seemed to characterize an immune signature of the *Diffuse* GC subtype considering the overlap with IFN-γ, KRAS, inflammatory response and allograft rejection (Figure 2C). Moreover, the invasive biology of the *Diffuse* type of GC could also be explained by the association of CD86, CIITA and IL-16 MR activity with the enrichment of IL6, JAK and STAT3 signaling. The overlap between the three MRs and the chr1q23 and chr7q34 cytogenetic bands (Supplementary Materials Figure S9), where localized genes are involved in lymphocyte activation and antigen presentation to T cells (CD84, CD48, CD1C) and gene coding for several T cell receptor variable regions, seemed to define an activation of the T cell-mediated response. In addition, CD86, IL16 and CIITA regulons intersected with several GO gene sets (Supplementary Materials Figure S12) confirming their role in T cell activity regulation and IFN-γ production. Finally, the CIITA regulon was significantly associated with MAML1 and ETS target genes (Supplementary Materials Figure S11), which were involved in the regulation of differentiation, survival and proliferation of lymphoid cells, even with the expression of cytokines and chemokines. On the contrary, in *Intestinal* GC, the regulons of the active MRs, FOXM1 and TOP2A, resulted in strongly associated hallmark and GO, relative to the mitotic spindle assembly, G_2_/M checkpoint and E2F gene sets (Figure 2D) together with Kang doxorubicin resistance and the Farmer breast cancer cluster 2 (proliferation and 8q amplicon genes) gene set, respectively (Supplementary Materials Figure S15). The HNF1 transcription factor gene set appeared enriched in *Intestinal* GC (Supplementary Materials Figure S16) while, beyond the 8q24 band (as expected from enriched the Farmer breast cancer cluster 2), the chrXq28 band resulted in significant association between the regulons of the SSX1, SSX4 and SOLHLH1 MRs, which are involved in stem cell maintenance and are indicated as cancer and testis antigens (Supplementary Materials Figure S14). By highlighting the biologically relevant gene networks active in the two histological types of GC, a distinct cancer cell phenotype was confirmed for *Diffuse* and *Intestinal* GCs. Moreover, several putative target genes that may modify the aberrant cell phenotype clearly emerged.

### 3.4. The Association of MRs Activity with Clinical Variables or Prognosis Confirms the Relevance of Underlying Molecular Profile

**Figure 3:**
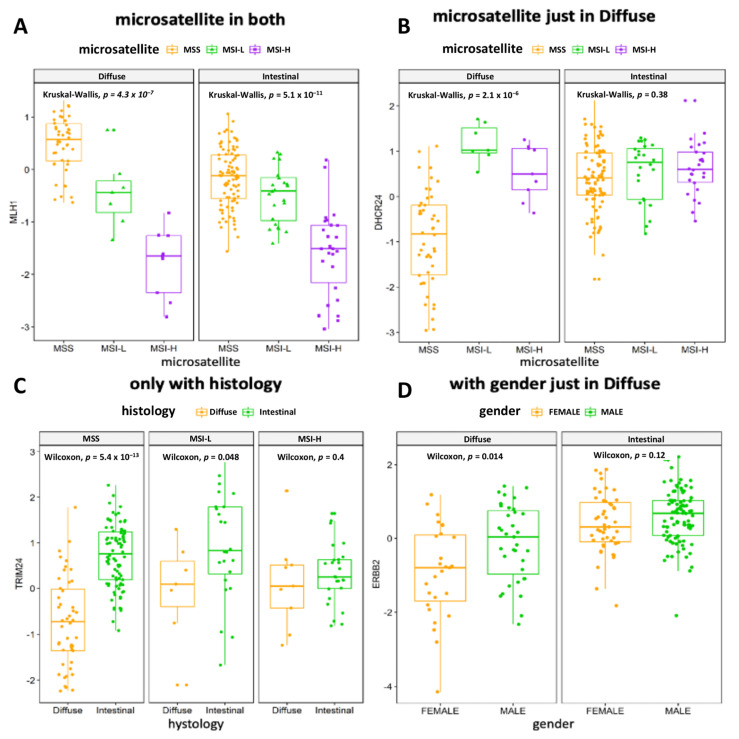
Boxplots of selected significant associated ssMRs NES and clinical features. (**A**–**C**) Relationships among microsatellite, histotype and different MRs. (**D**) Highlight of a gender-associated MR in the *Diffuse* subgroup. NES on the y-axis examples is how much regulon is active. Wilcoxon test informs the association of the clinical feature with MR.

Although the two histological subgroups investigated here are widely recognized in clinical practice, their consistency is weakened by molecular and clinical heterogeneity. We aimed to highlight the more homogeneous subnetworks for each subtype and pinpoint subnetworks related to patient outcomes and tumor features, even if not related to the histological division. Accordingly, we highlighted some interesting MR examples. We performed an ssGSEA and obtained the grade of activation or deactivation (NES) for each gene set in each sample by identifying those MRs capable of influencing patient outcomes and GC natural history. The heatmap in Supplementary Materials Figure S19 depicts the profile of the top five MRs in *Diffuse* and *Intestinal* GCs for each sample. Thanks to this analysis, we can pinpoint the MRs that more homogeneously associate with the *Diffuse* or *Intestinal* groups or molecularly identify different samples, which may be candidates for a different classification or treatment strategy. To characterize the activity of each MR, we investigated, with a linear model, the association between histological and molecular characteristics. Meaningful results (F-test *p*-value < 0.05) are reported in Supplementary Materials File S5. As proof of concept, we explored some interesting results given to the community in a useful dataset to generate hypotheses. The MLH1 NES strongly associated with the microsatellite status in both *Diffuse* and *Intestinal* GC samples as depicted in Figure 3A, being a factor independent from histology.

**Figure 4:**
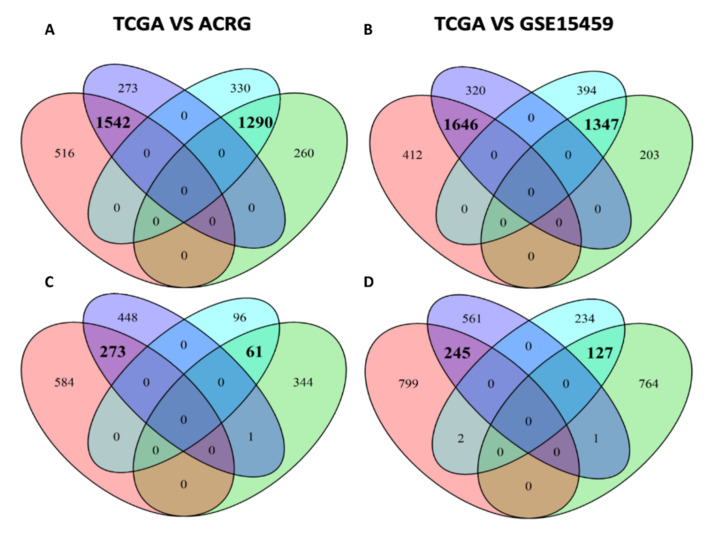
Venn diagrams showing the overlap between the TCGA and the validation dataset data. (**A**) The overlap of the DEGs between *Diffuse* vs. *Intestinal* subgroups in TCGA and ACRG dataset. (**B**) The overlap of the DEGs between *Diffuse* vs. *Intestinal* subgroups in TCGA and GSE15459 dataset. (**C**) The overlap of the MRs between *Diffuse* vs. *Intestinal* subgroups in TCGA and ACRG dataset. (**D**) The overlap of the MRs between *Diffuse* vs. *Intestinal* subgroups in TCGA and GSE15459 dataset. All overlaps in bold are statistically significant (*p*-vaule < 0.01).

Differences between the two subgroups can also be appreciated, as is the case of MR DHCR24 in Figure 3B, which was significantly associated with microsatellite status in the *Diffuse* but not *Intestinal* samples. Moreover, in Figure 3C, we can appreciate the case of the MR TRIM24 as very differently active in *Intestinal* vs. *Diffuse* GC only in the MSS samples. These examples showed the possibility of exploring the association of an MR with any clinical feature when its behavior is homogeneous or different between subgroups or is different among feature levels. Lastly, in Figure 3D, the MR ERBB2 results were significantly associated with gender in the *Diffuse* but not *Intestinal* samples, suggesting a putative gender- and histology-driven rethink of treatment strategies. Using the Cox proportional-hazards model, we described the change in OS, DSS, DFS or PFI if the NES of the MRs rose by one unit. In Supplementary Materials File S6, we report all the significant results (*p*-value < 0.05). Interestingly, the MR CGB5, having the higher hazard ratio (HR) considering OS, DSS and PFI results, was associated with a worse prognosis while the MR ARHGAP33 results, having the lower HR considering DSS and PFI and the second lowest by OS, were associated with a better prognosis. For each patient and for every additional NES unit of the MR CGB5, the risk of death, disease-specific death and disease progression increased by 79% (HR 1.79), 152% (HR 2.52) and 112% (HR 2.12), respectively. On the contrary, for each patient and every additional NES unit of the MR ARHGAP33, the risk of death, death by disease and disease progression fell by 30% (HR 0.70), 44% (HR 0.56) and 48% (HR 0.52), respectively. When we looked at the Cox proportional-hazards model results, focusing on the MRs previously highlighted, it was very easy to pinpoint the HR of the VGLL3, INHBA and PRRX1 regulons, 27% (HR 1.27), 44% (HR 1.44) and 46% (HR 1.46). The worst prognosis, related to a higher activity of regulons, was associated (*p*-value << 0.001) with *Diffuse* tumors and a greater tumor mass (TNM:T). In addition, among the worst prognosis of MRs associated with EMT, there was SNAI2 (OS HR: 1.51 and DSS HR: 1.52). ILF3 regulon was associated with a better prognosis (DFI HR 0.53 and PFI HR 0.75) and with high activity in *Intestinal* tumors (*p*-value << 0.001). Moreover, the ACIN1 (an apoptosis-related gene) regulon (see Supplementary Materials Figure S20) was associated with a better prognosis (OS HR 0.73 and DSS HR 0.68 and PFI HR 0.67) and with high activity in *Intestinal* tumors (*p*-value < 0.001). According to Townson SM et al. [40], the SAFB gene acts as a negative regulator of cell proliferation. This evidence corroborated our result showing that the high activity of the SAFB regulon (see Supplementary Materials Figure S21) in *Intestinal* tumors was strongly associated (*p*-value << 0.001) with a better prognosis (OS HR 0.78, DSS HR 0.73 and PFI HR 0.78). Finally, our results were validated in the ACRG and GSE15459 cohort, by performing differential gene expression analyses on GSA and MRA in *Diffuse* and *Intestinal* GCs. We reported, as Venn diagrams, the overlaps among the DEGs (Figure 4A,B) and MRs (Figure 4C,D). 

In Table 3, we report the amount of overlapping enrichments between TCGA-STAD and each validation dataset.

Moreover, 59 MRs associated with the OS by Cox analysis in the TCGA dataset were confirmed in both ARCG and GSE15459 (Supplementary Materials File S7). Among them, there are some of the biologically characterized *Diffuse* MRs like SNAI2, ANTXR1, PRRX1, EYA4, VGLL3, AEBP1, PDGFRB, THY1, LZTS1 and FSTL1. Using this approach, we identified a set of clinically relevant MRs that more homogeneously associated with the *Diffuse* or *Intestinal* type of GC or associated with a known clinical feature. These data may be useful to characterize patients, addressing them to a different classification or treatment strategy.

## 4. Discussion

Despite its decreasing incidence, GC remains one of the most common causes of death for neoplasm worldwide. Recent epidemiological trends indicate a relative increase in the rate of the *Diffuse* histotype, especially in western countries [41,42,43,44,45]. The biological bases of the *Diffuse* type of GC behavior are still poorly characterized and, in particular, a precise signaling network reconstruction is lacking. In this work, we aimed to highlight the molecular determinants that characterize the two main histological GC subtypes by generating a biological network-based model. We first investigated the relations among the histological groups and the other clinicopathological data, finding a relevant association with the pathologic stage, the TNM:N and therapy outcomes. Moreover, by multivariate Cox proportional hazards analysis, we found that histology and pathologic stages were independent factors influencing overall survival. Since several histological groups were numerically too small for suitable analyses, we divided the samples into two transcriptionally homogeneous groups: *Diffuse* (poorly cohesive, not SRC) and *Intestinal* (tubular and iNOS). We highlighted that there was no significant association between these groups and clinicopathological data as well as survival. According to this, the clinical and survival differences reported above could be imputed to the SRC and non-tubular *Intestinal* groups. Subsequently, we deployed a computational pipeline aimed to highlight the key genes, networks and pathways characterizing the two groups. Differential expression analysis produced two refined lists of GC histology-related candidate biomarkers. Enrichment analyses, as well as master regulator analyses, highlighted a greater number of activated gene sets and regulons in *Diffuse* than in *Intestinal* GC. The MRs, by their nature, represented a robust way to describe key genes and network features of a characterized group. The regulons, being data-inferred subnetworks, might have a cryptical biological meaning so the overlap with significantly enriched gene sets strongly hints about their function as a biological labeler. This step allowed us to select activated subnetworks showing literature-derived, biologically interesting features but retained possibly related non-literature-derived gene connections. The ssMRA allowed us to investigate the homogeneity of the subgroups’ MRs, pinpointing which MR might be a better candidate for this classification and which one could lead to a different classification or treatment strategy. As matter of fact, the activity of many MRs showed an association with survival while there was no difference between the *Diffuse* and *Intestinal* subgroups, hinting at a possible different classification. Interestingly, two independent datasets validated many of the results confirming the goodness of the computational pipeline and showing the robustness of the MR analysis across different datasets. It is important to remember that the MR represents an entire network (the regulon), suggesting the underlying molecular explanation of the observed biological behavior. For example, the Cox proportional-hazards model associates the MR VGLL3 with a worse prognosis and the Fisher test associates *VGLL3* with *Diffuse* tumors and a greater tumor mass (TNM:T), according to a previous report [39]. In this way, we pinpointed the field of investigation for possible subsequent studies because we suggested a wide, biologically coherent network, rather than a single gene associated with survival. Indeed, we speculated that the negative impact of VGLL3 activity on a patient’s prognosis could be related to its role in modulating EMT, collagen synthesis and extracellular matrix organization as highlighted by the intersection of the gene network and several gene sets. Its biological activity has also been demonstrated in vitro by Hori N et al., who described the promotion of an EMT-like phenotype and an increased motility in VGLL3-expressing lung cancer cells [46]. SNAI2 is another enriched MR. It is a prototypical epithelial-to-mesenchymal transition transcriptional factor, which promotes the loss of cell adhesion and polarity, conferring a migratory and invasive phenotype. SNAI2 is also indicated as a molecular determinant of cancer stem cell behavior and therapy resistance [47]. 

Our study showed that the *Diffuse* type of GC was also characterized by the dysregulation of immune signaling that, according to Hill et al., created a favorable microenvironment promoting tumor progression, invasiveness, angiogenesis and metastasis [48]. The role of MRs involved in this crosstalk between cancer and immune cells can be dual either modulating or stimulating the immune system. Indeed, CD86 is known to bind CTLA-4 to inhibit antigen presenting cells and the activation of T cells [49]. CIITA and IL16 promote immune cell activation and a pro-inflammatory microenvironment that can compromise the integrity of the gastric epithelium. It has been demonstrated that CIITA promotes the expression of MHC class II, which favor CD8+ T cell activation, and that its expression is induced by IFN-γ [9,50,51], a significantly enriched hallmark that we found in the *Diffuse* type of GC. Interestingly, IFN-γ seemed to also be a driver of disease progression during chronic gastritis to metaplasia by direct killing gastric parietal cells [52]. Similarly, IL-16 activity was found to be associated with disease progression in many cancer types, including those of the gastrointestinal tract [53]. A pro-inflammatory microenvironment can also be explained by the enrichment of the KRAS signaling hallmark, since it includes several genes that code for chemokines and cytokines (LIF, CXCL10, etc.), which overlap with those in the other immune hallmarks. The heatmaps evidencing the association of MRs with GOs, pathways and other gene sets enriched in *Diffuse* or *Intestinal* GC subtypes remark the biological features outlined by hallmarks. The overlap between the three MRs highly enriched in *Diffuse* GC and positional gene sets seemed to define an activation of the T cell-mediated response. Indeed, in the chr1q23 cytogenetic band localized genes were involved in lymphocyte activation and antigen presentation to T cells (CD84, CD48, CD1C). On the other hand, *Intestinal* GC was characterized by MRs (such as FOXM1, TOP2A and CENPF) involved in mitotic spindle assembly, G2/M checkpoint regulation and expression of E2F target genes, suggesting a proliferative phenotype. The association between a proliferative GC subtype and *Intestinal* histology was previously reported to be characterized by the upregulation of the centromeric family of proteins (CENPs), among them CENPF [54]. Authors demonstrated that CENPs, in particular CENP-I, promoted cell proliferation and migration, apoptosis inhibition and EMT and is associated with the TP53 mutation. Interestingly, ATM and p53 regulated FOXM1 expression via E2F in Epirubicin resistant breast cancer [55], highlighting a possible connection between CENPF, FOXM1 and E2F regulons. The FOXM1 regulon also showed great overlap with Kang doxorubicin resistance gene set. Recently, Qi W. et al. demonstrated that the regulation of the level of ERBB2 in the gastric cancer cell lines, accordingly produced a change in the expression of FOXM1, indicating that the expression level of FOXM1 was at least partially regulated by ERBB2 [56]. Interestingly, Qi W. et al. also revealed that FOXM1 and ERBB2 expression was associated with poor survival. Similarly, the E2F family of transcription factors (E2Fs) regulated many cellular processes, canonically cell cycles but also angiogenesis, DNA damage response, apoptosis and drug resistance both in cancer and cancer stem cells [34]. According to these findings, in *Intestinal* GC, the chrXq28 band resulted in significantly associated activity between SSX1, SSX4 and SOLHLH1 MRs, which are involved in stem cell maintenance and are indicated as cancer and testis antigens [57]. The enriched positional gene set included genes that seemed to play a role in embryonic development [58], GC transformation and progression (the MAGEA gene family) [59] and telomere maintenance (DKC1) [60]. Interestingly, this cytogenetic band included genes of the PRAME regulon that is known to confer a growth advantage to cancer cells [61,62]. In addition, *Intestinal* GC enrichment of HNF1 transcription factor target motifs suggested a marked transcriptional activity and modulation of cholesterol and sterol homeostasis, essential for cell division [63]. HNF1 proteins also regulate the embryonic development of gastrointestinal tract organs [64].

## 5. Conclusions

Our results showed that distinct biological features characterize *Diffuse* and *Intestinal* gastric cancers, suggesting molecular bases for clinicopathological differences between the two histotypes. In particular, *Diffuse* GC is characterized by the alteration of pathways involved in immune cell regulation and extracellular matrix organization, while the *Intestinal* type is associated with impairments to the cell cycle regulation pathways and alteration of response to growth factors and hormones. Overall, the histological differences between *Diffuse* and *Intestinal* GC are based on genetic and epigenetic factors and neither of these two ways can be ignored. Many studies have shown that unveiling the molecular complexity of cancer can help predict specific cancer biomarkers (diagnostic, prognostic and drug-susceptibility) and design biological network-based anti-cancer therapies [7,22,23] by shifting from a single-gene to a gene-network personalized therapy approach. The characterization of these signaling networks could also lead to the identification of targets aimed to improve anti-tumor immunity and overcome the immune escape mechanisms of cancer cells. This is because gene regulatory networks can help to resolve key issues in cancer research by reflecting information from multiple regulatory levels.

## Figures and Tables

**Table 1:** Patient characteristics and clinicopathological data.

Characteristics		Histological Type	
		Diffuse (*n* = 62)	Intestinal (*n* = 140)
Age (years, median with range)		61.5 (53–70)	67 (58–72)
Gender	Woman	27 (44%)	47 (34%)
	Man	35 (56%)	93 (66%)
Anatomic	Antrum Distal	31 (51%)	50 (37%)
	Cardia Proximal	5 (8%)	18 (13%)
	Fundus Body	22 (36%)	55 (40%)
	Gastroesophageal Junction	3 (4.8%)	13 (10%)
	Missing	1	4
Anatomic JGCA (Japanese Gastric Cancer Association)	Distal	31 (53%)	50 (41%)
	Proximal	27 (47%)	73 (59%)
	Missing	4	17
Pathologic stage	I	5 (8%)	21 (15%)
	II	18 (31%)	25 (18%)
	III	31 (53%)	70 (52%)
	IV	5 (8%)	21 (15%)
	Missing	3	3
Pathologic T	T1	0 (0%)	10 (7%)
	T2	16 (26%)	28 (20%)
	T3	23 (37%)	64 (46%)
	T4	23 (37%)	38 (27%)
	Missing	0	33
Pathologic N	N0	13 (21%)	33 (24%)
	N1	18 (29%)	34 (25%)
	N2	15 (24%)	42 (31%)
	N3	16 (26%)	27 (20%)
	Missing	0	4
Pathologic M	M0	54 (90%)	125 (91%)
	M1	6 (10%)	12 (9%)
	Missing	2	3
Microsatellite status	MSS	46 (74%)	90 (64%)
	MSI.L	7 (11%)	24 (17%)
	MSI.H	9 (15%)	26 (19%)
	Missing	0	0
Primary therapy outcome success	Complete Response	31 (55%)	74 (63%)
	Partial Response	1 (2%)	3 (3%)
	Stable Disease	7 (12%)	11 (9%)
	Progression Disease	17 (30%)	29 (25%)
	Missing	6	23

NOS: not otherwise specified; JCGA: Japanese gastric cancer association; MSS: microsatellite stable; MSI.L: microsatellite instable low; MSI.H: microsatellite instable high; CR: complete response; PR: partial response; SD: stable disease; PD: progressive disease. Percentages, given as histology, are among each characteristic without the missing samples.

**Table 2:** Summary of the significantly enriched gene sets in *Diffuse* and *Intestinal* GC samples.

	Diffuse		Intestinal	
	N. Out of TOT	%	N. Out of TOT	%
Hallmarks	12/50	24%	6/50	12%
GOs	775/10,192	7.6%	184/10,192	1.8%
Pathways	926/5529	16.7%	257/5529	4.6%
Chromosome positions	6/299	2%	9/299	3%
Motifs/miRNAs	550/3735	14.7%	6/3735	0.16%
Immunologic signature	958/4872	19.6%	79/4872	1.6%

**Table 3:** Summary of the differentially enriched categories between *Diffuse* and *Intestinal* GC in TCGA, ARCG and GSE15459 dataset. Statistically significant (*p*-value < 0.01) overlaps are in bold.

	TCGA	ARCG	GSE15459	TCGA vs. ARCG	TCGA vs. GSE15459
Enriched in Diffuse					
hallmark	12	8	6	7 *	5 *
Go	926	837	675	522 *	301 *
Pathways	775	942	925	566 *	452 *
Motif	550	1089	1762	391*	480 *
Chromosome positions	6	4	4	1	0
Enriched in Intestinal					
hallmark	6	7	6	5 *	4 *
GO	184	133	313	62 *	82 *
Pathways	257	400	912	184 *	209 *
Motif	6	6	44	0	2 *
Chromosome positions	9	2	3	1	1

* Statistically significant (*p*-value < 0.01) overlaps.

## Data Availability

Data was obtained from the TGCA, GTEx and GEO repositories [third party] and are available at https://xenabrowser.net/datapages/ (accessed on 15 June 2021) and at https://www.ncbi.nlm.nih.gov/geo/ (accessed on 15 June 2021).

## Supplementary Materials

The following supporting information can be downloaded at: https://www.mdpi.com/article/10.3390/cancers14194961/s1. Figure S1: Plot of the significant association between the histological subgroups and, stage and TNM:N categories in TCGA STAD. Figures S2–S5: Forest plot of the stepwise selected clinical features associated with the survival by multivariate Cox analysis. Figure S6: Plot of the significant association between the revised histological subgroups, stage and TNM:N categories in TCGA STAD. Figure S7: Venn diagram of the upregulated genes in *Diffuse* vs. all the *Intestinal* samples and the two most abundant *Intestinal* (tubular and NOS) subgroups in the TCGA STAD dataset. Figure S8: Workflow. Figures S9–S18: HMs of the overlaps between regulons and ontologies. Figure S19: Heatmap of the top five *Diffuse* and *Intestinal* MRs. Figure S20: Heatmap of ACIN1 regulon associated with a better prognosis and high activity in *Intestinal* tumors. Figure S21: Heatmap of SAFB regulon associated with a better prognosis and high activity in *Intestinal* tumors. Table S1: Summary of the GC clinical data. File S1: Differentially expressed genes. File S2: Ontologies enriched in *Diffuse* tumor. File S3: Ontologies enriched in *Intestinal* tumor. File S4: Master regulators differentially active between *Diffuse* vs. *Intestinal*. File S5: Histological and molecular characteristics associated with master regulators. File S6: Survival associated with MR’s NES. File S7: Survival associated with MR’s NES in validation dataset.
