# Supplementary material for: Gene Regulatory Network Characterization of Gastric Cancer’s Histological Subtypes: Distinctive Biological and Clinically Relevant Master Regulators
Source: Cancers (Basel). 2022 Oct 10;14(19):4961. doi: 10.3390/cancers14194961 (PMC9563962; doi:10.3390/cancers14194961)
Supplement: Supplementary file 1 [file cancers-14-04961-s001.zip › Supplementary Figures&Tables.pdf]

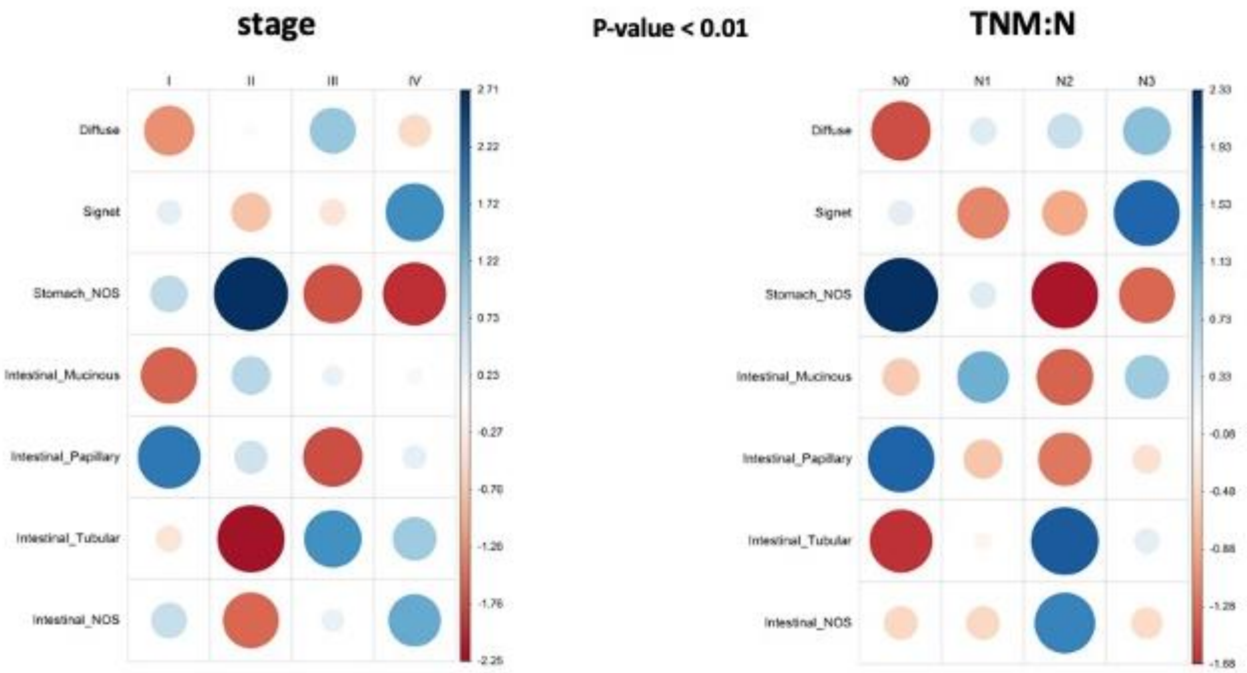

Figure S1. Plot of the significant association between the histological subgroups and, stage and TNM:N categories in TCGA STAD. The size and the color intensity of the circles are proportional to the absolute association. Positive associations are in blue while negative in red.

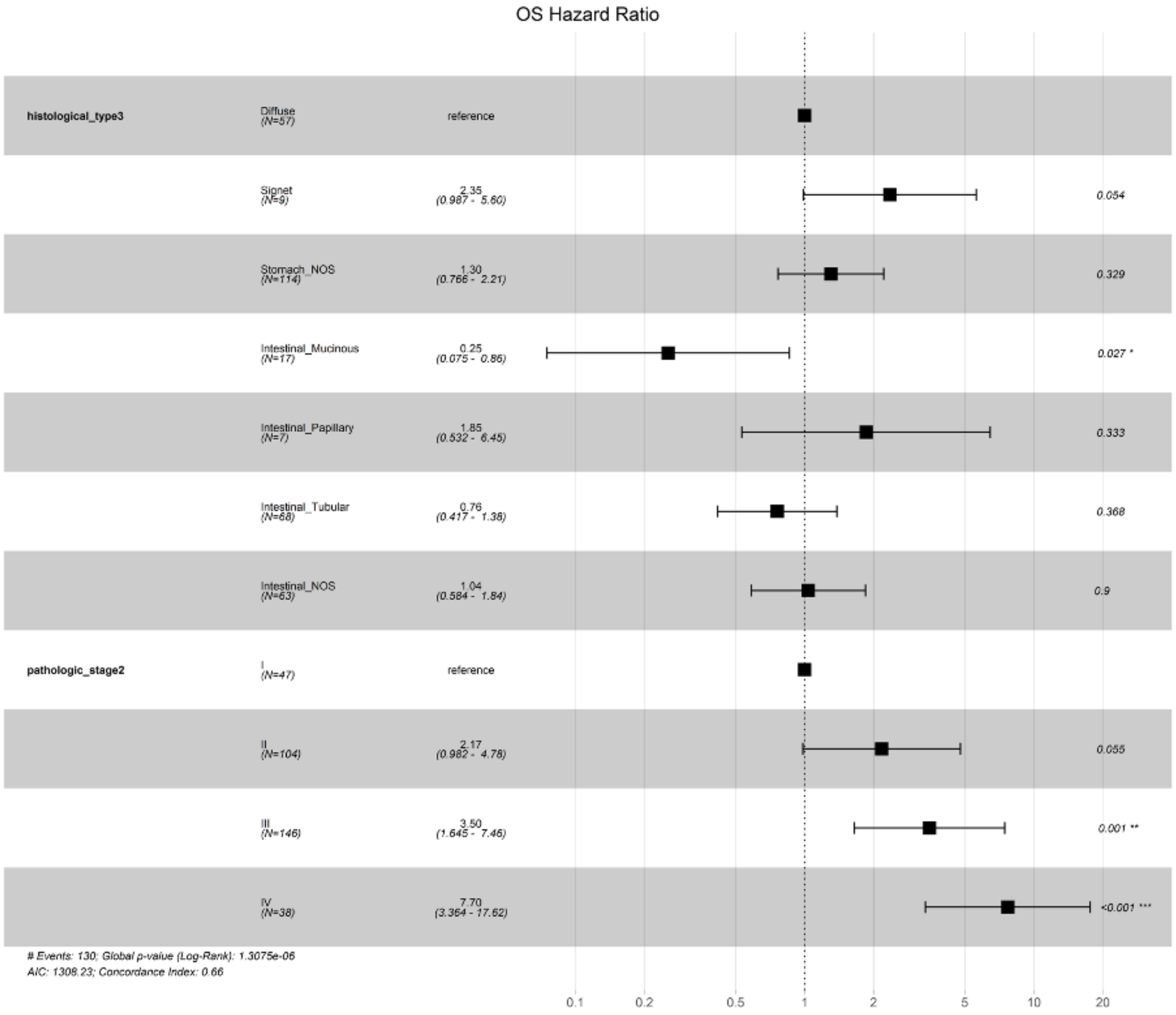

Figure S2. Multivariate Cox Proportional-Hazards Model. Overall survival.

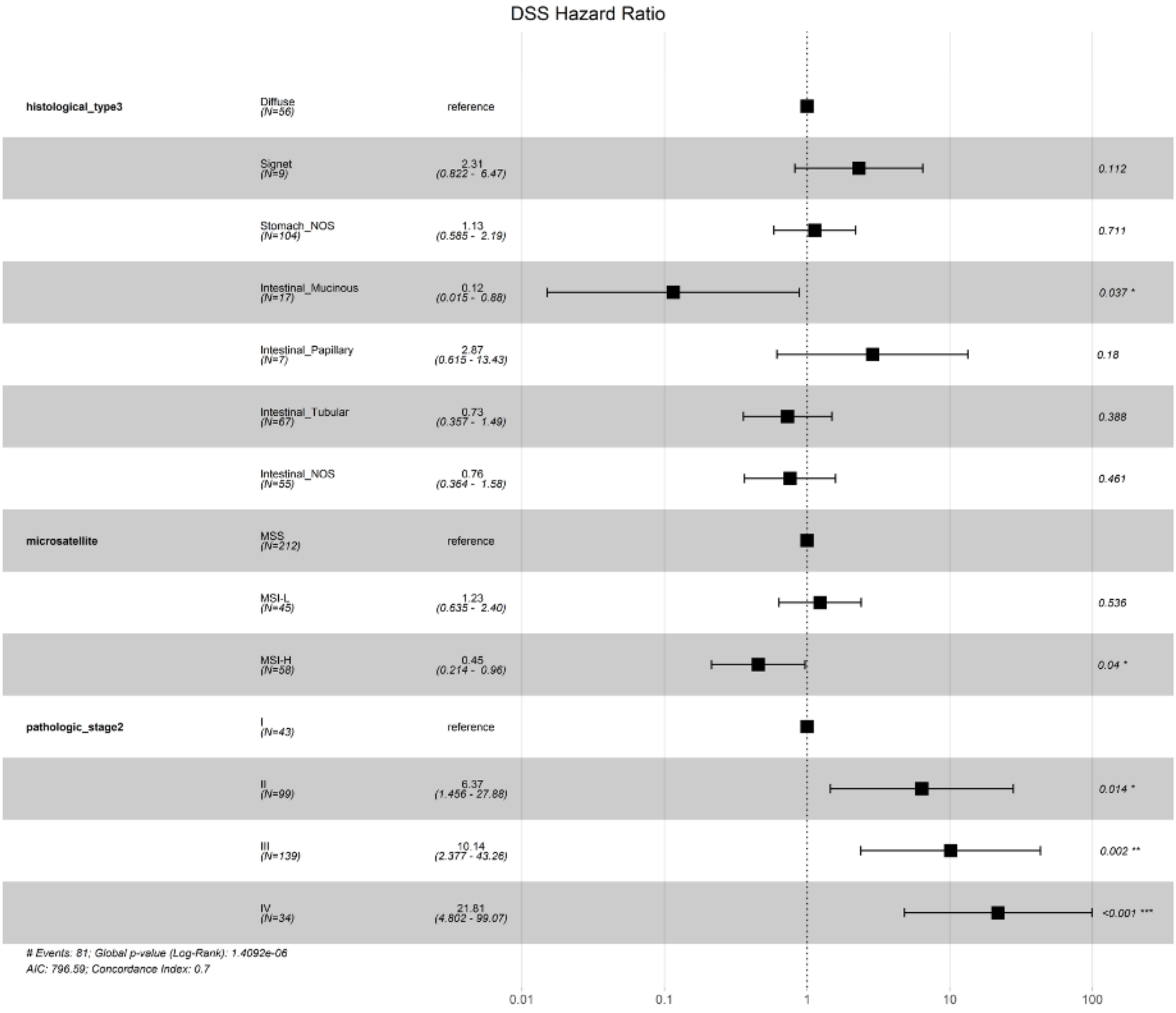

Figure S3. Multivariate Cox Proportional-Hazards Model. Disease specific survival

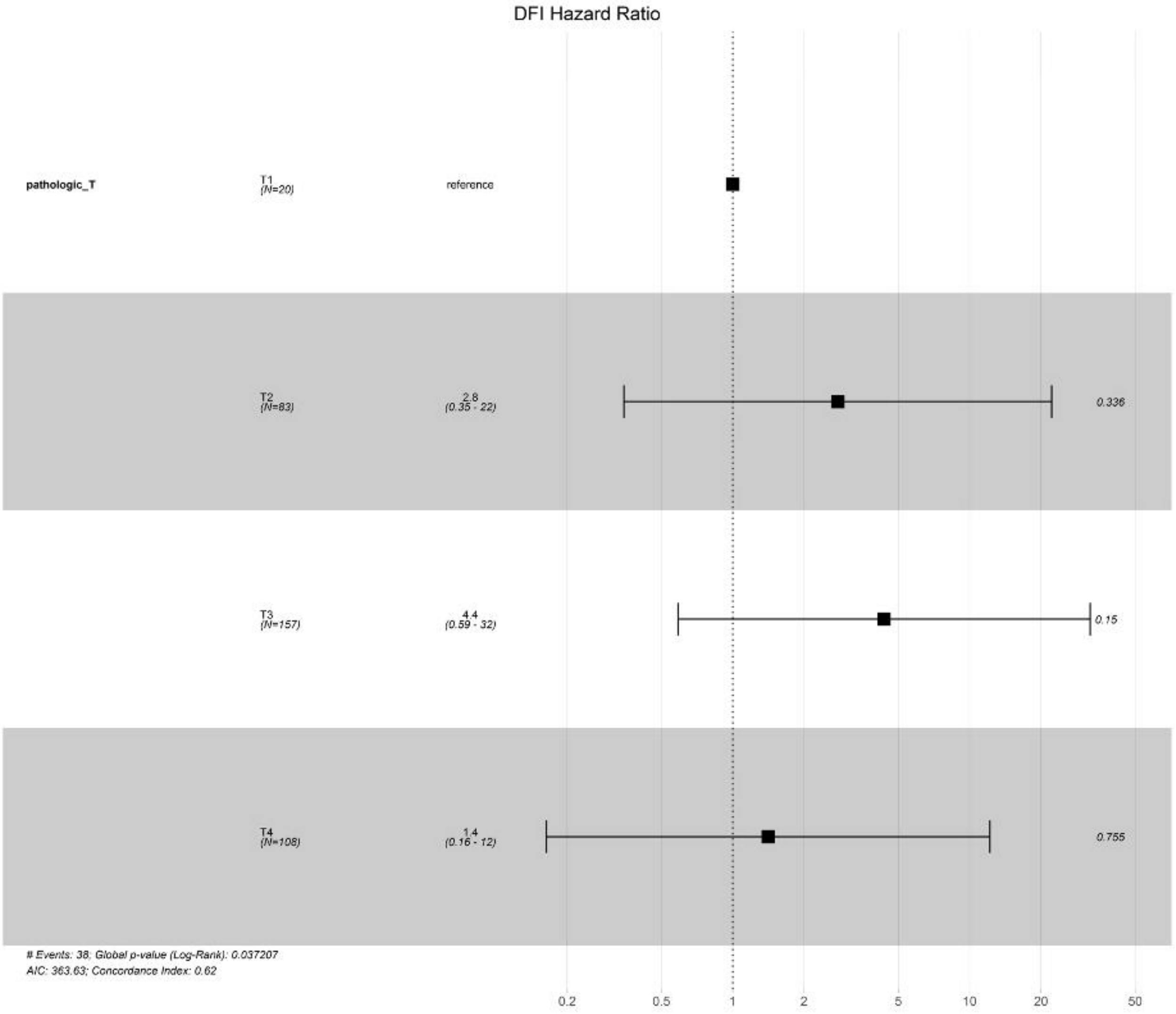

Figure S4. Multivariate Cox Proportional-Hazards Model. Disease free interval

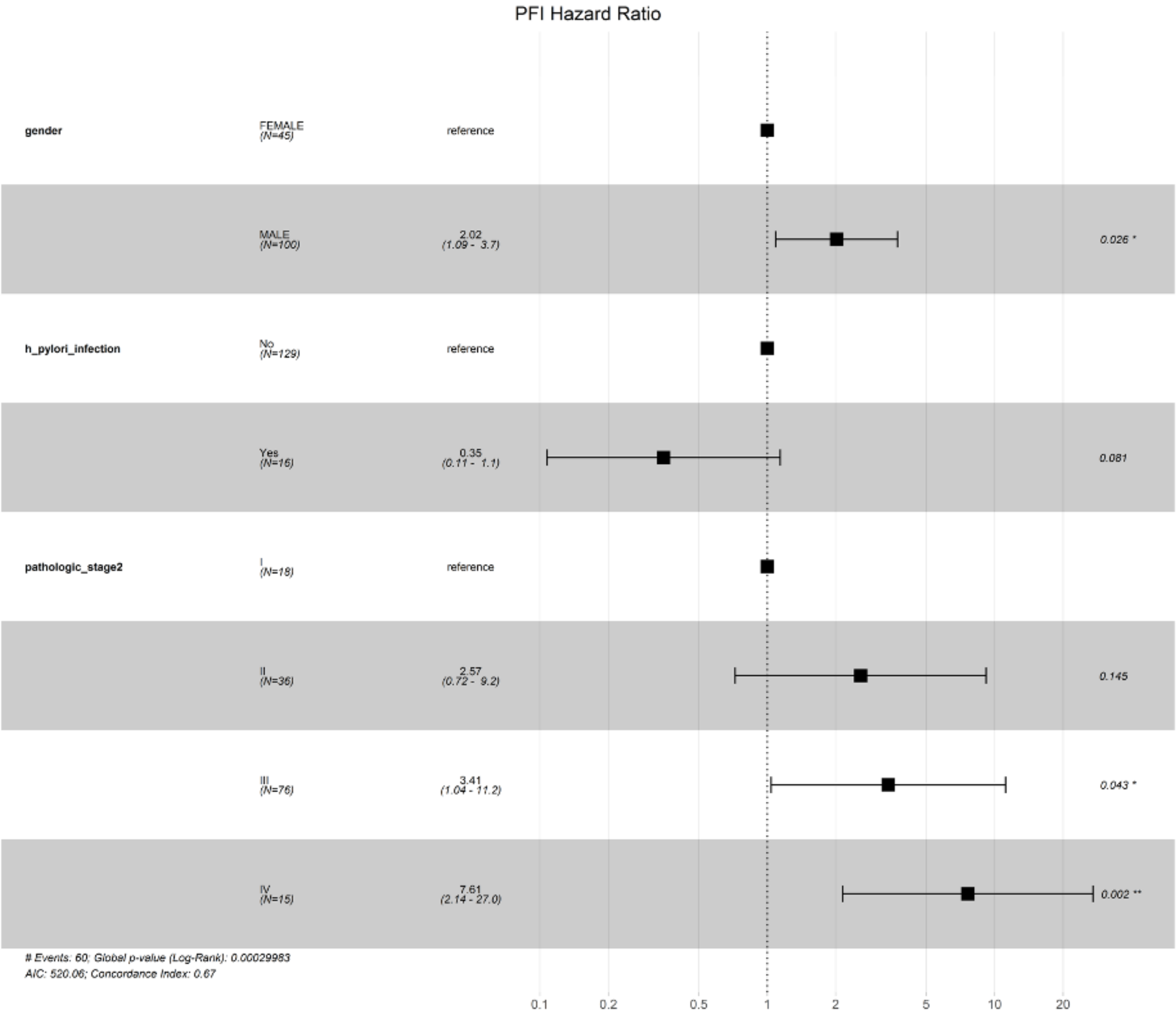

Figure S5. Multivariate Cox Proportional-Hazards Model. Progression free interval

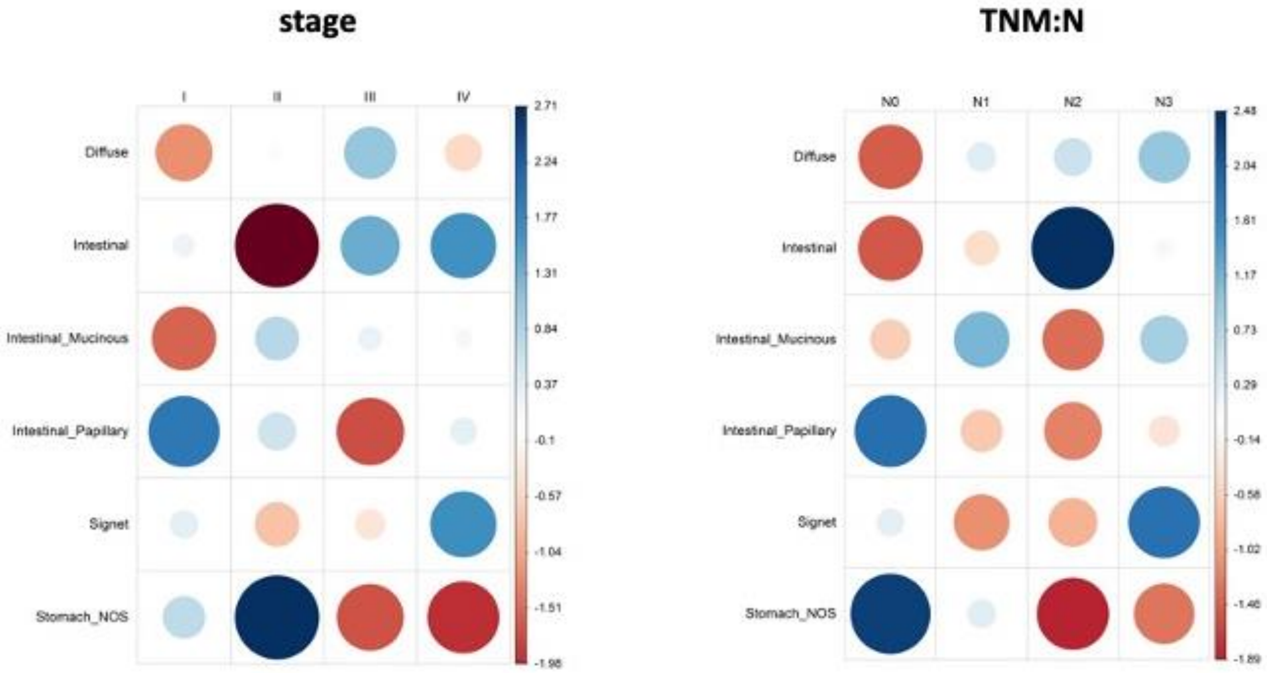

Figure S6. Plot of the significant association between the new defined histological subgroups and, stage and TNM:N categories in TCGA STAD. The size and the color intensity of the circles are proportional to the absolute association. Positive associations are in blue while negative in red.

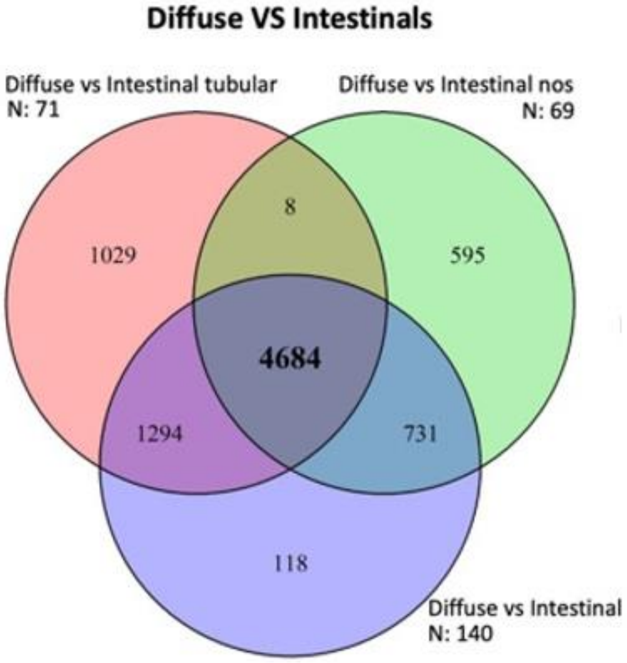

Figure S7. Venn diagram of the up-regulated genes in Diffuse vs all the Intestinal samples and the two most abundant Intestinal (tubular and NOS) subgroups in the TCGA STAD dataset.

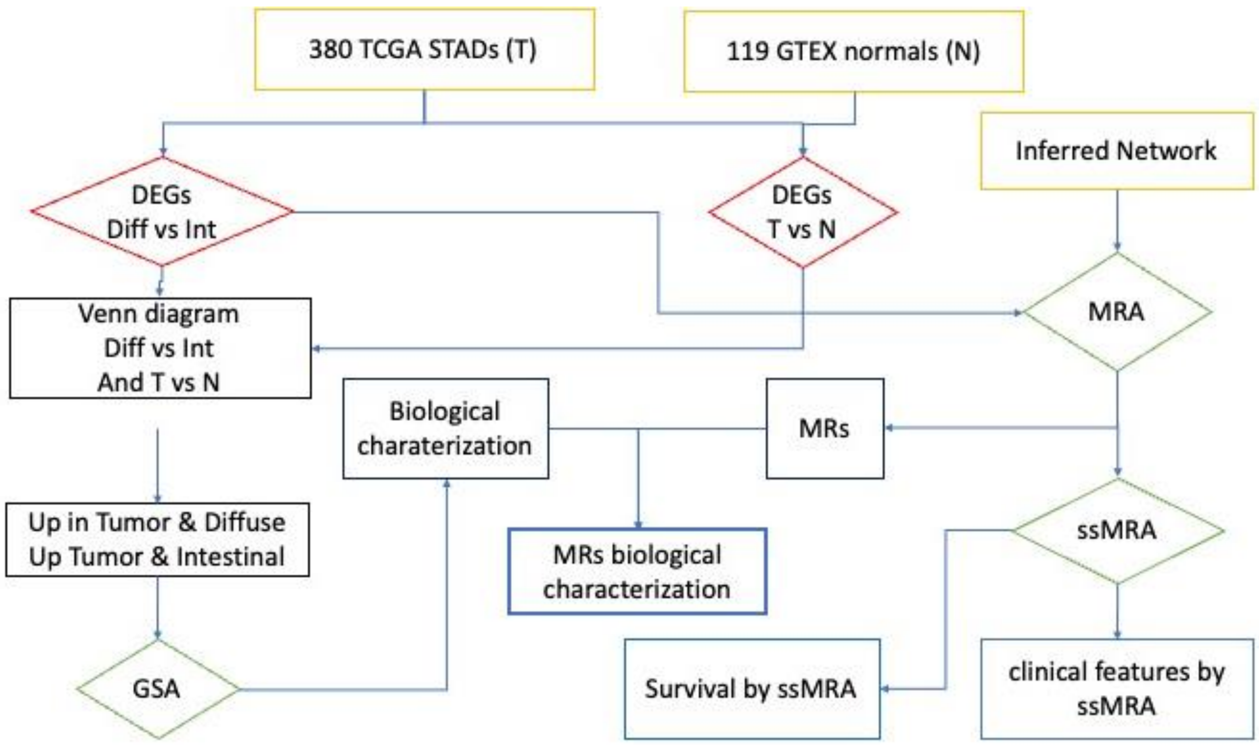

Figure S8. Workflow. STAD: stomach adenocarcinomas; normals: healty mucosa. Diff: diffuse GC; Int: Intestinal GC; Cox: Cox Proportional-Hazards Model; DEGs: differentially expressed genes; MR: Master regulator; MRA: Master Regulator Analysis; ssMRA: single sample Master Regulator Analysis; GSA: Gene Set Enrichment.

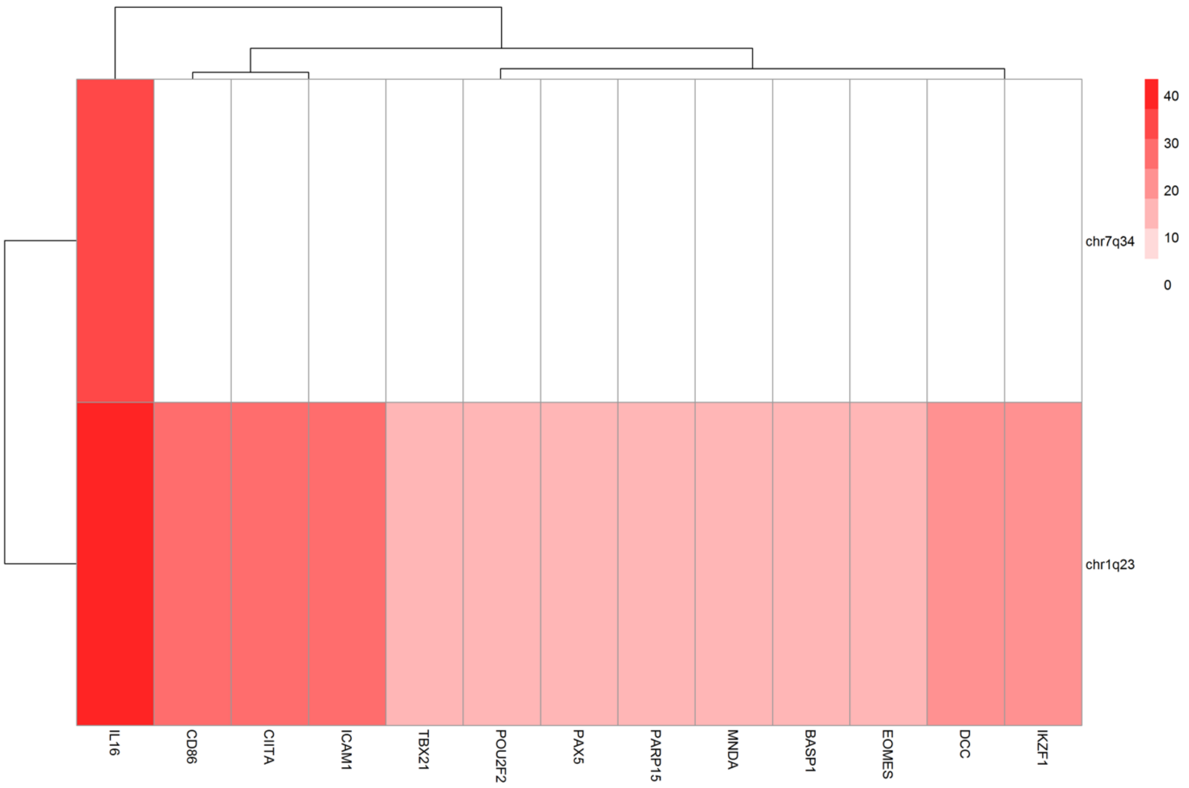

Figure S9. Heatmap of the top overlap between Diffuse MRs and chromosomal position gene sets. Deeper the red, greater the overlap.

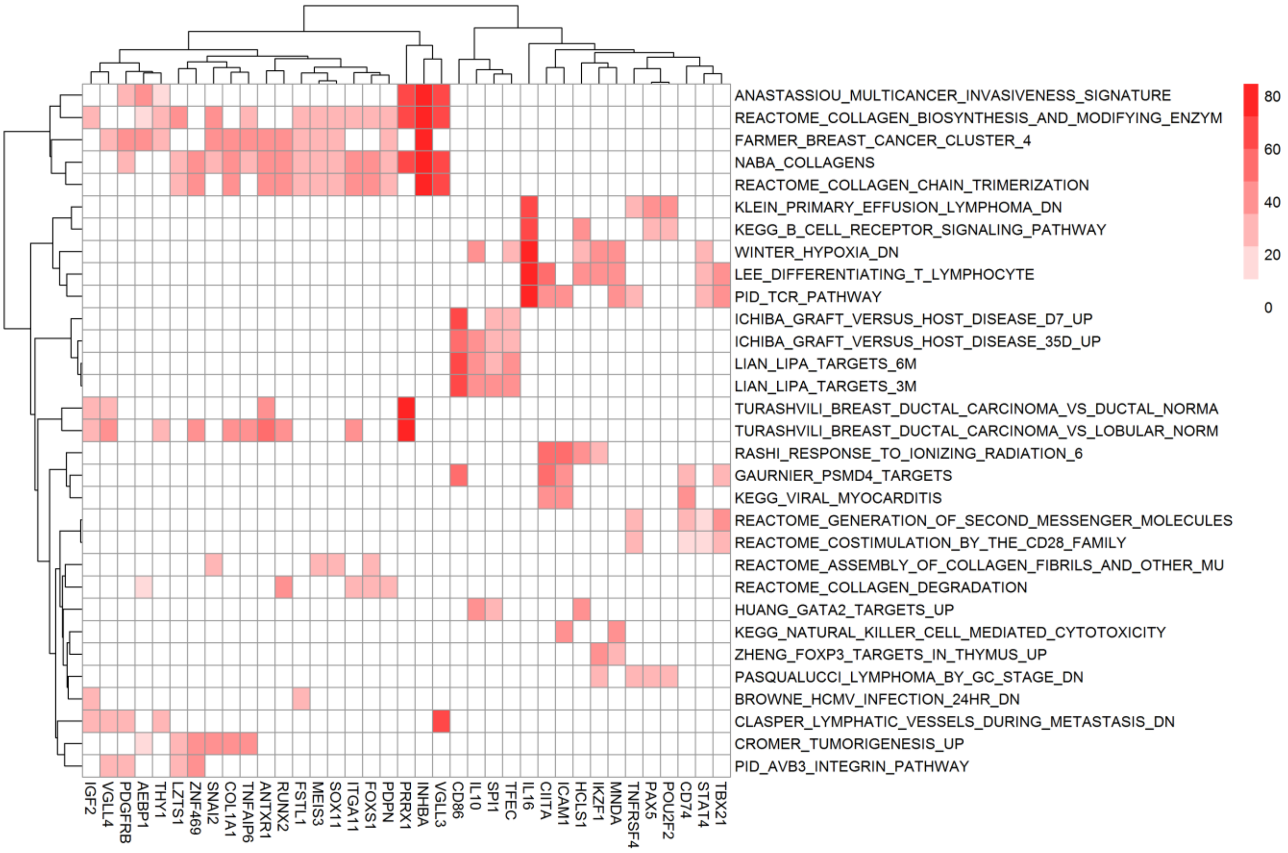

Figure S10. Heatmap of the top overlap between Diffuse MRs and Pathways. Deeper the red, greater the overlap

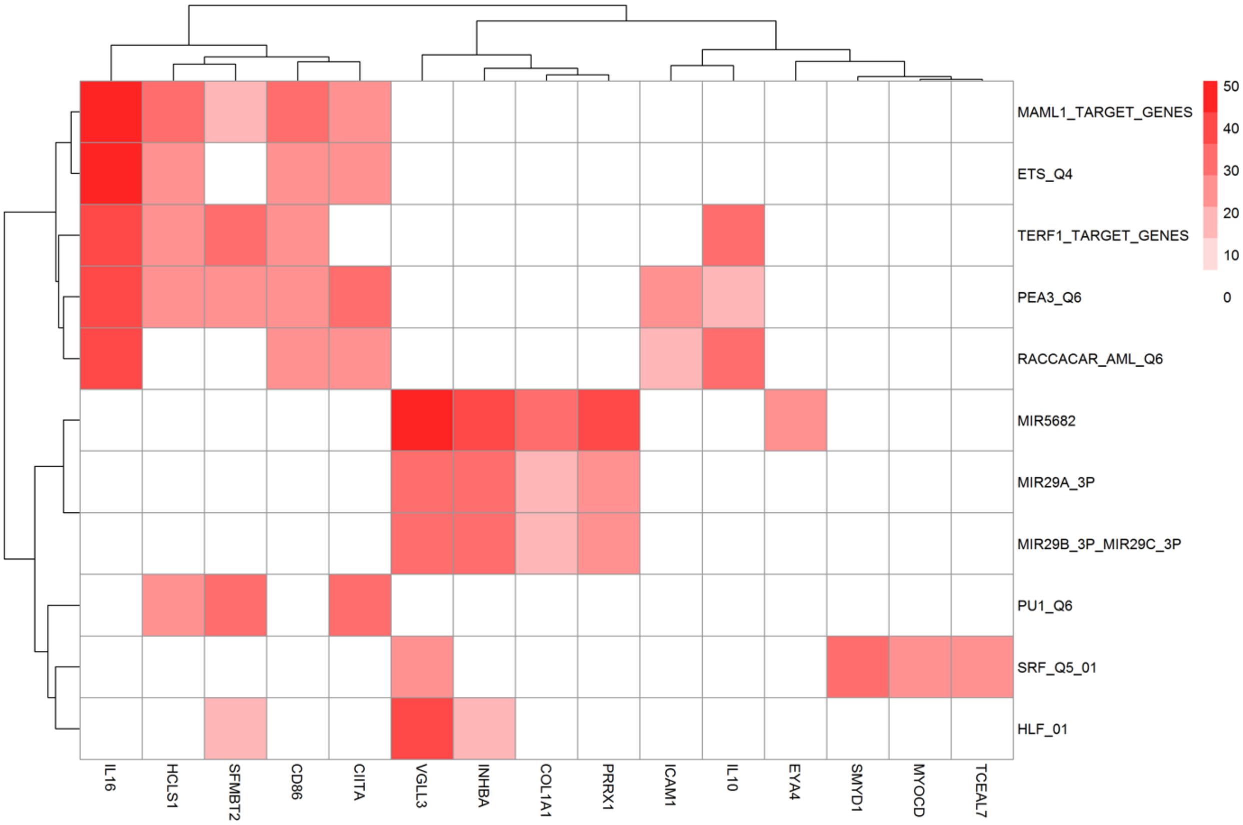

Figure S11. Heatmap of the top overlap between Diffuse MRs and, Motif and miRNAs. Deeper the red, greater the overlap

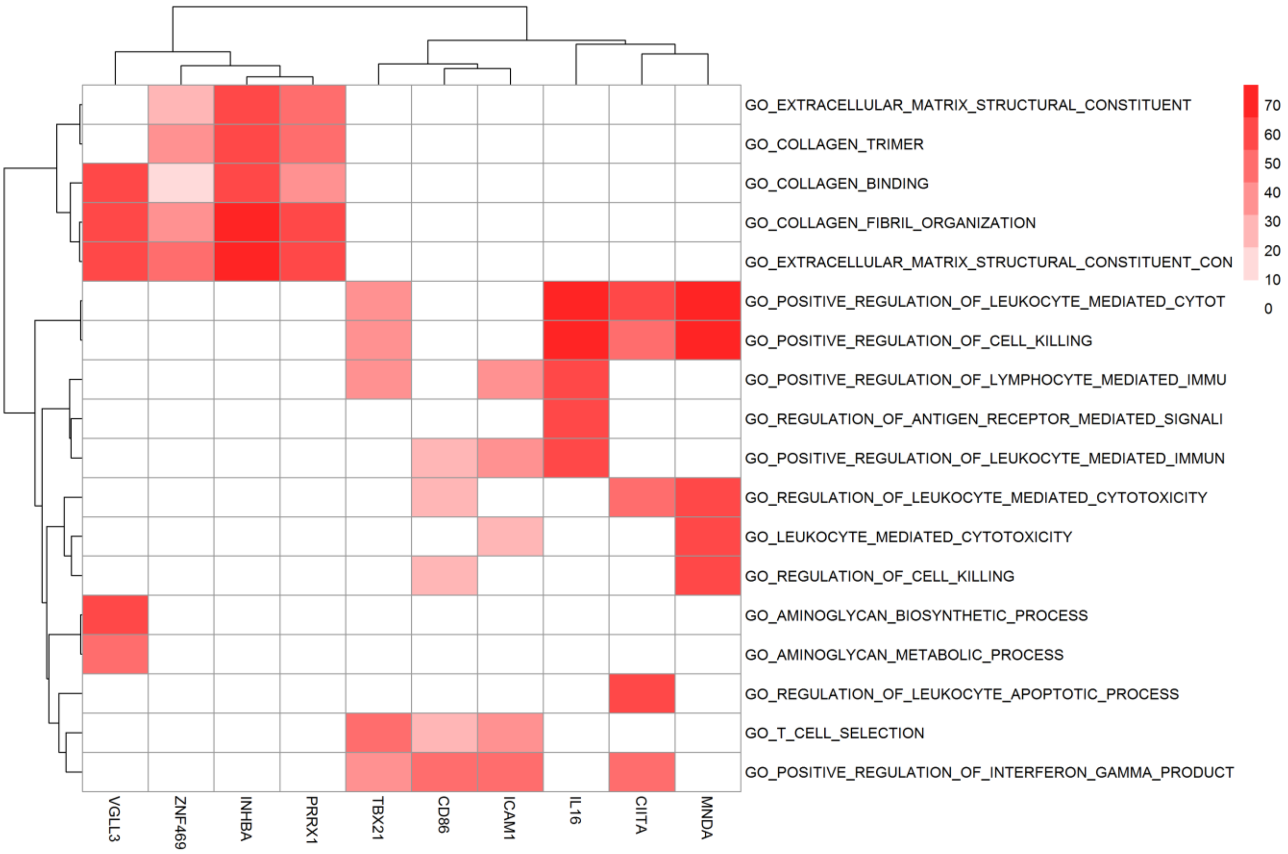

Figure S12. Heatmap of the top overlap between Diffuse MRs and GO. Deeper the red, greater the overlap

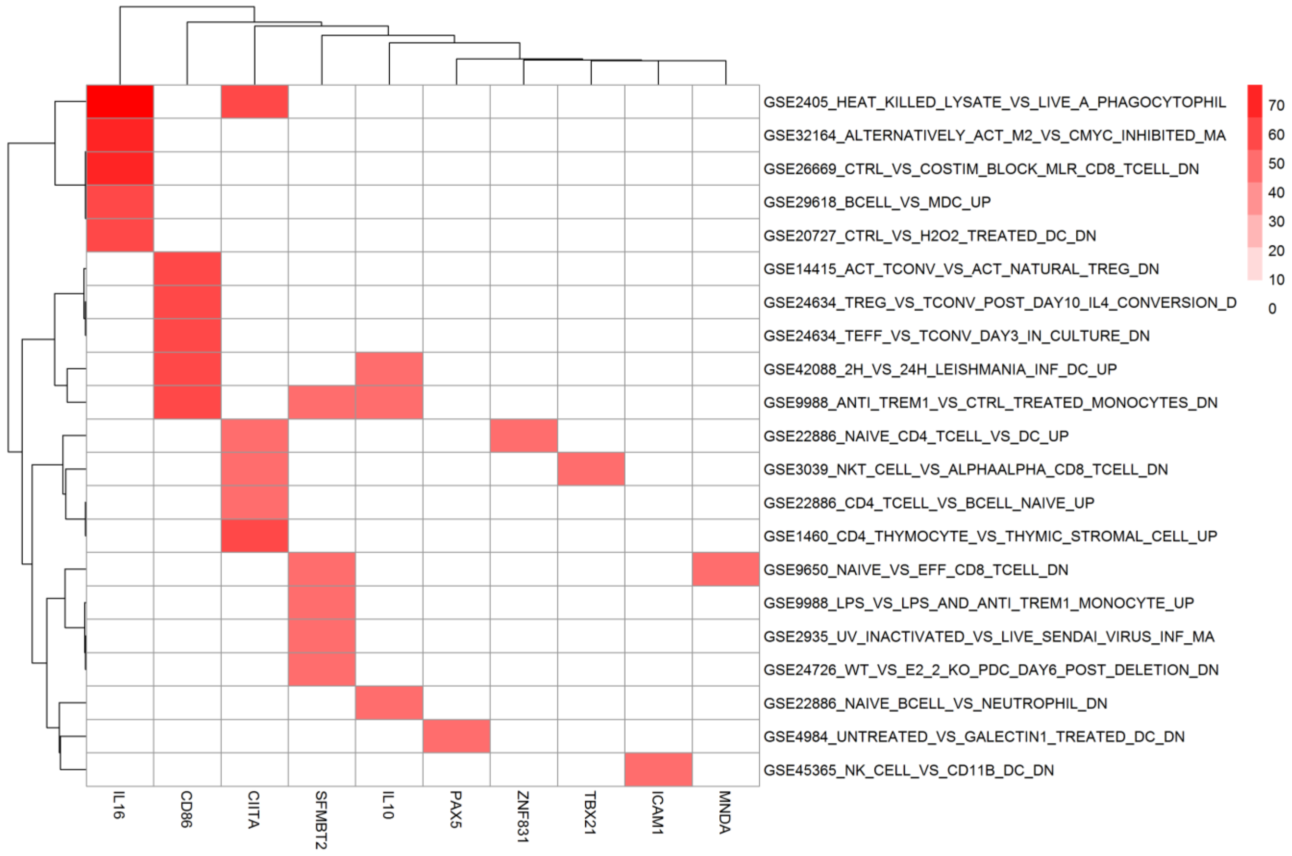

Figure S13. Heatmap of the top overlap between Diffuse MRs and Immune gene sets. Deeper the red, greater the overlap

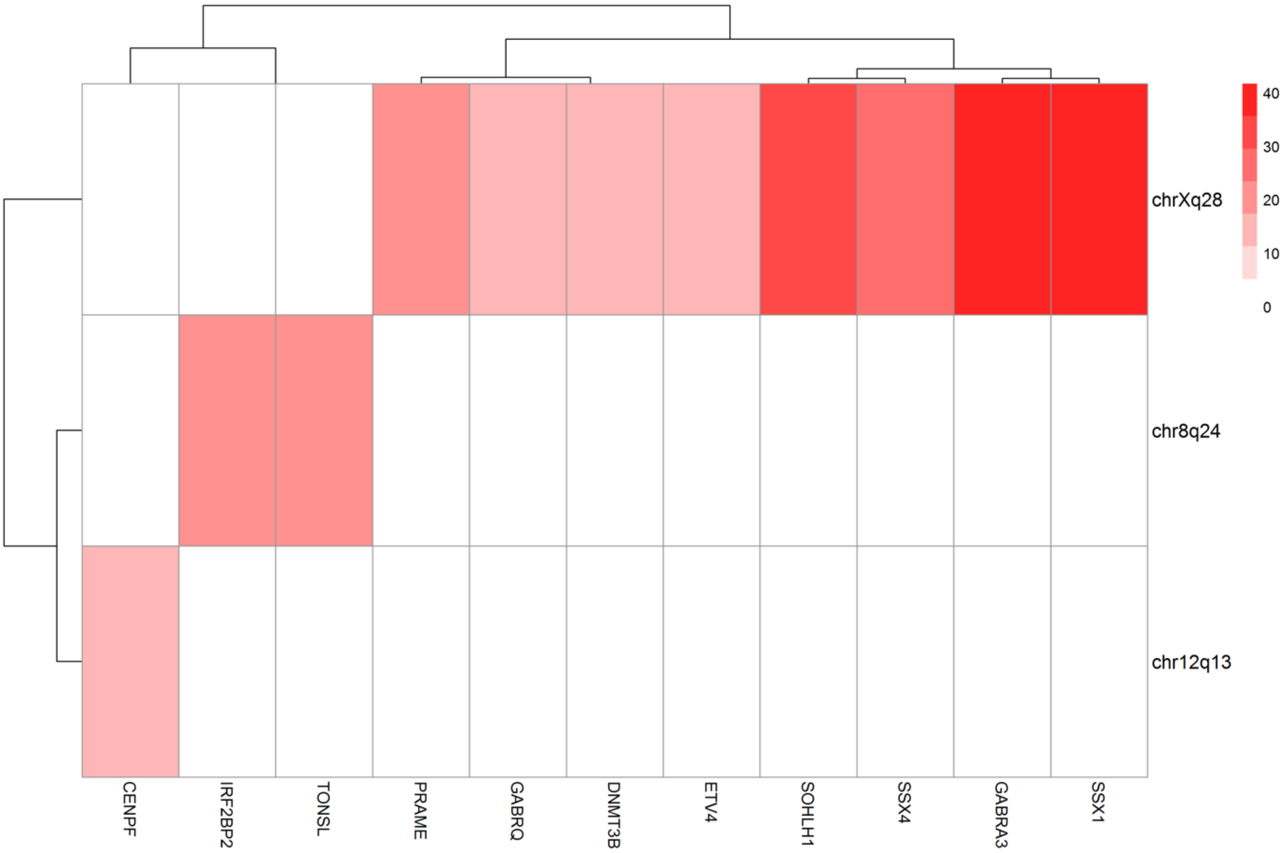

Figure S14. Heatmap of the top overlap between Intestinal MRs and chromosomal position gene sets. Deeper the red, greater the overlap.

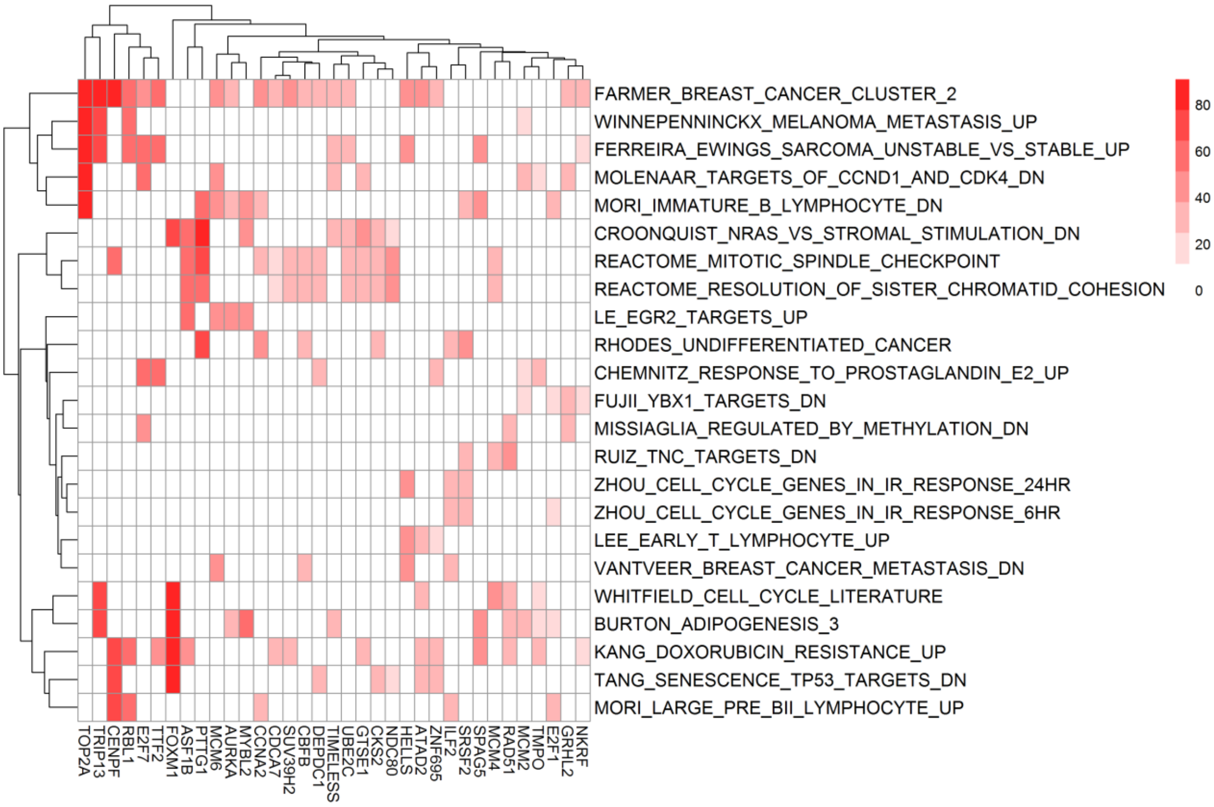

Figure S15. Heatmap of the top overlap between Intestinal MRs and Pathways. Deeper the red, greater the overlap.

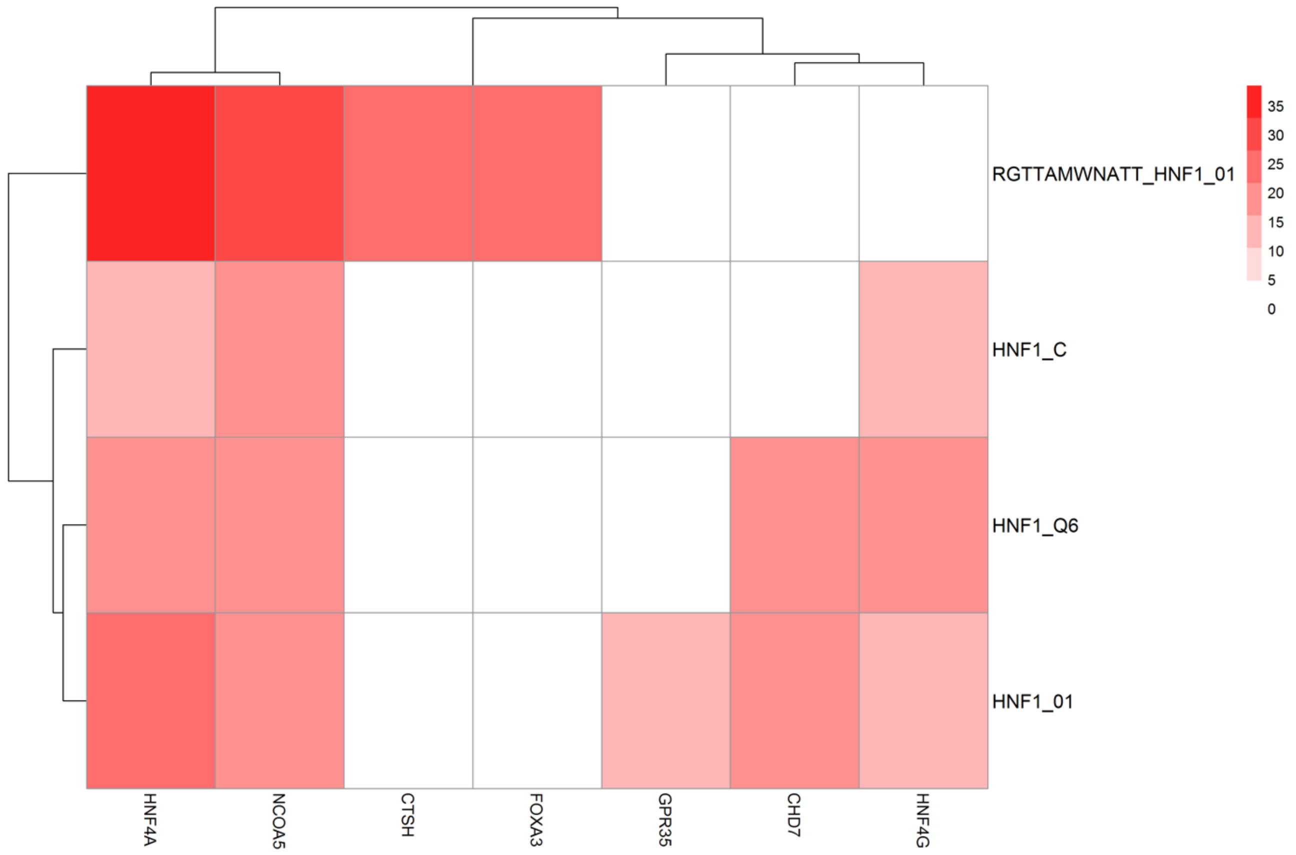

Figure S16. Heatmap of the top overlap between Intestinal MRs and, Motifs and miRNAs. Deeper the red, greater the overlap

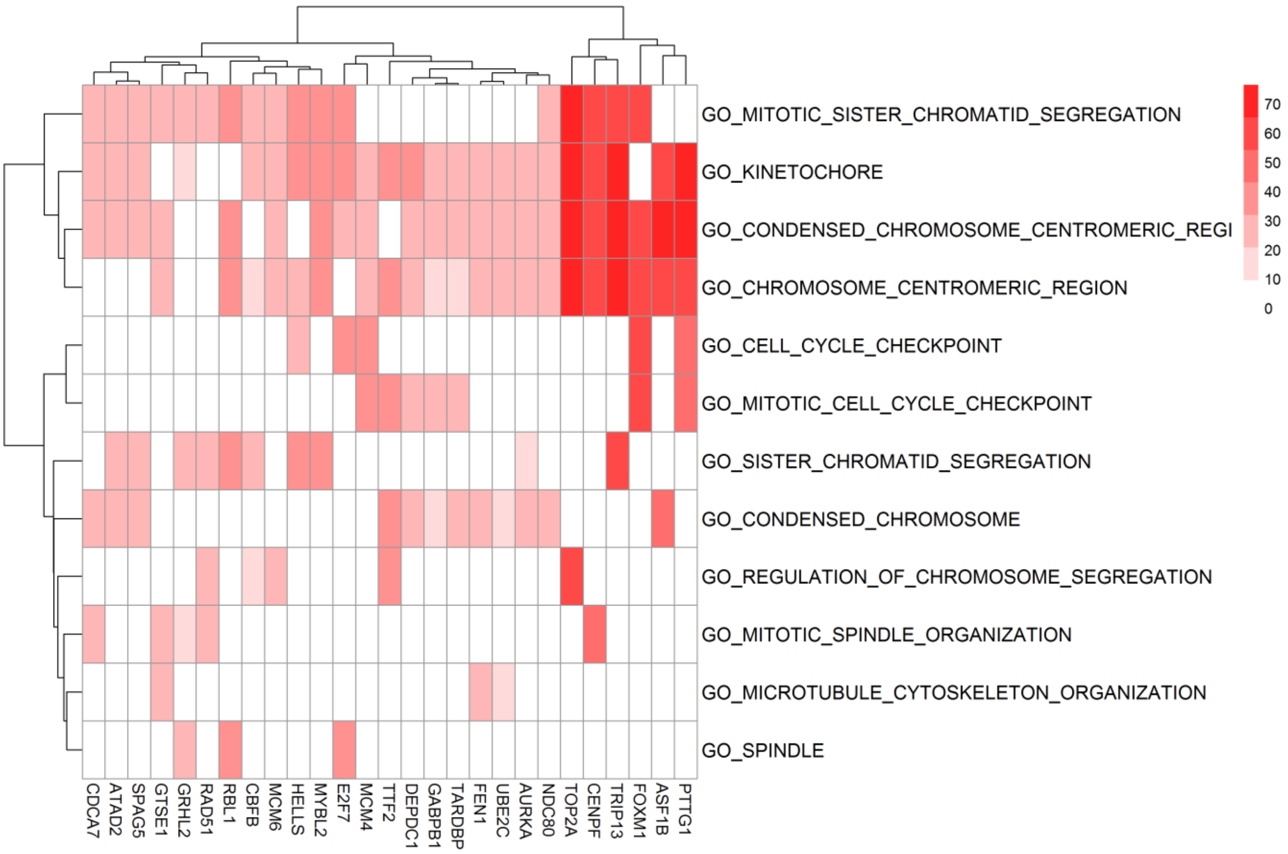

Figure S17. Heatmap of the top overlap between Intestinal MRs and GO. Deeper the red, greater the overlap.

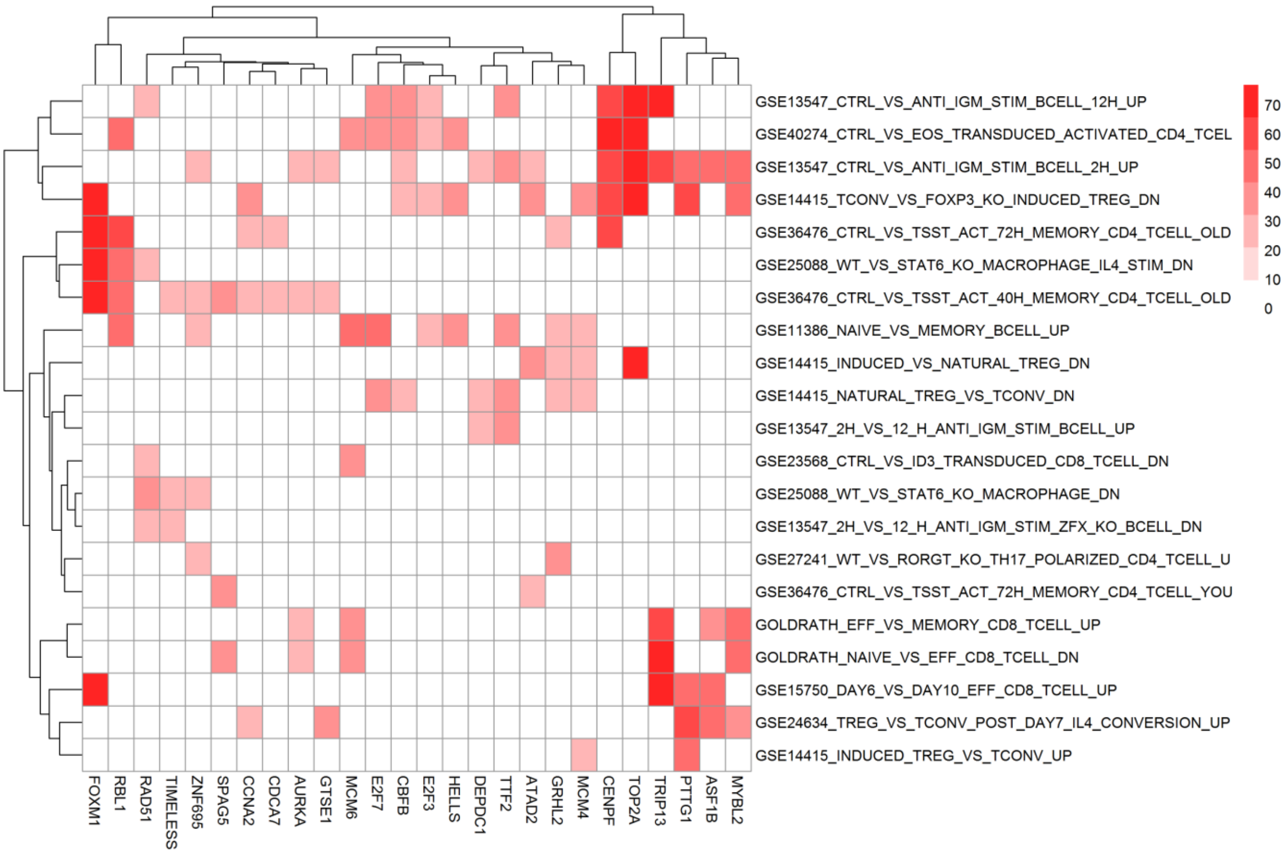

Figure S18. Heatmap of the top overlap between Intestinal MRs and immune gene sets. Deeper the red, greater the overlap

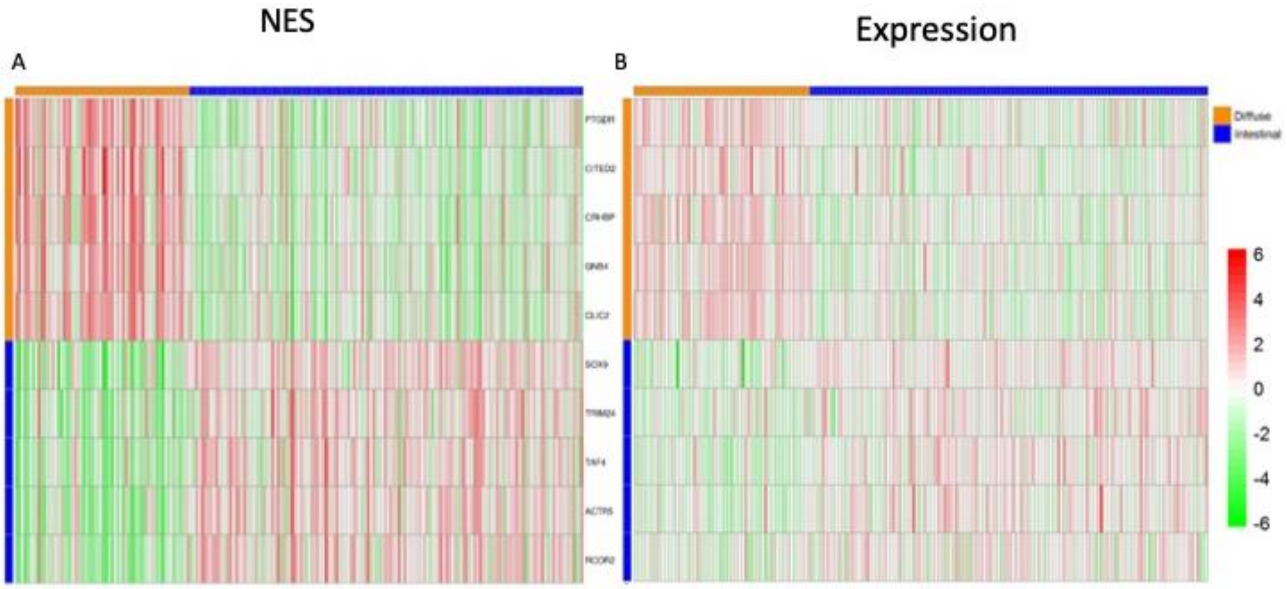

Figure S19. Heatmap of the top 5 Diffuse and Intestinal MRs. NES and expression in red-green color scale. In legend, above the HM, Diffuse samples in orange while Intestinal samples in blue. On the left Diffuse MR in orange while Intestinal MR in blue.

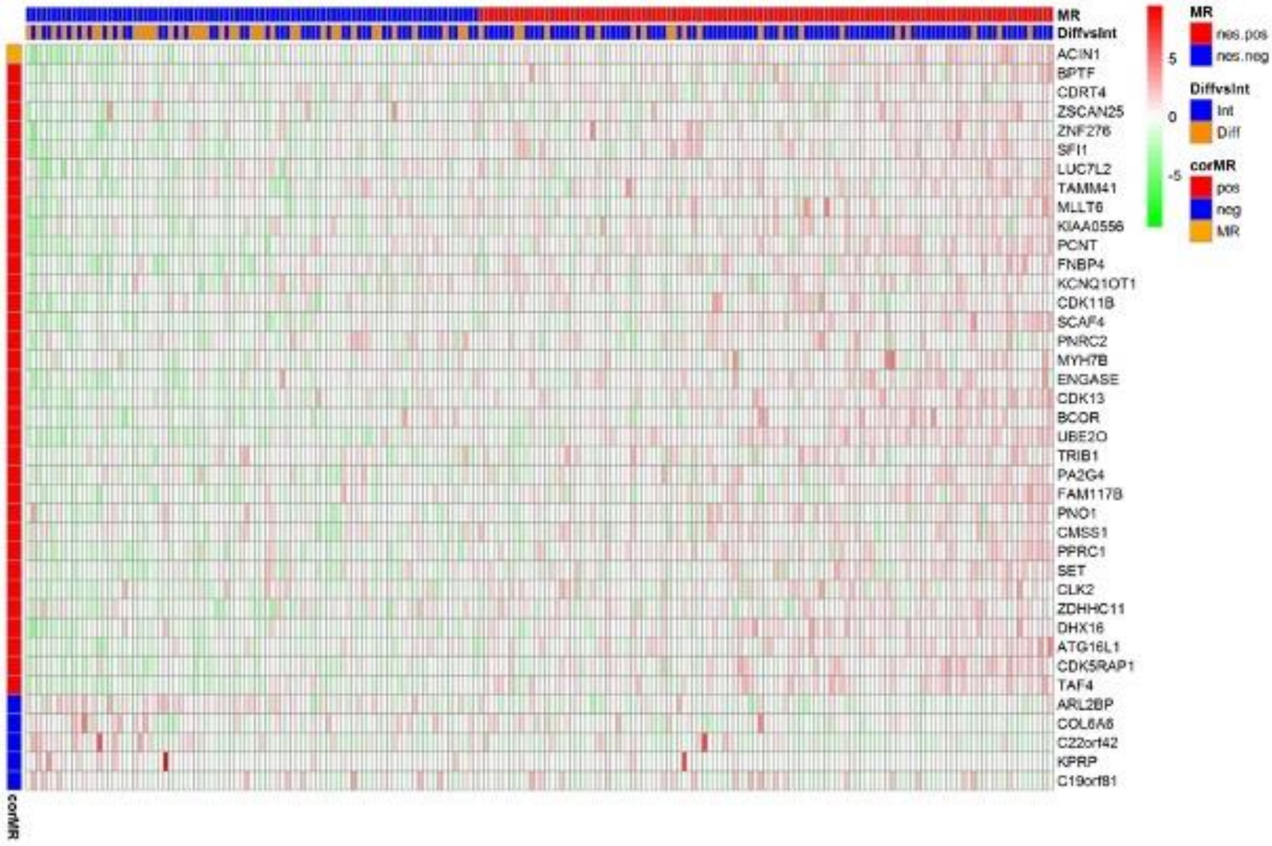

Figure S20. Heatmap of ACIN1 regulon associated with a better prognosis and with high activity in Intestinal tumors. Expression in red-green color scale. In legend, above the HM, Diffuse samples in orange while Intestinal samples in blue; in red samples with positive ACIN1 NES, in blue with negative ACIN1 NES. On the left of the HM Diffuse MR in orange, genes with negative correlation in blue and the MR expression in orange.

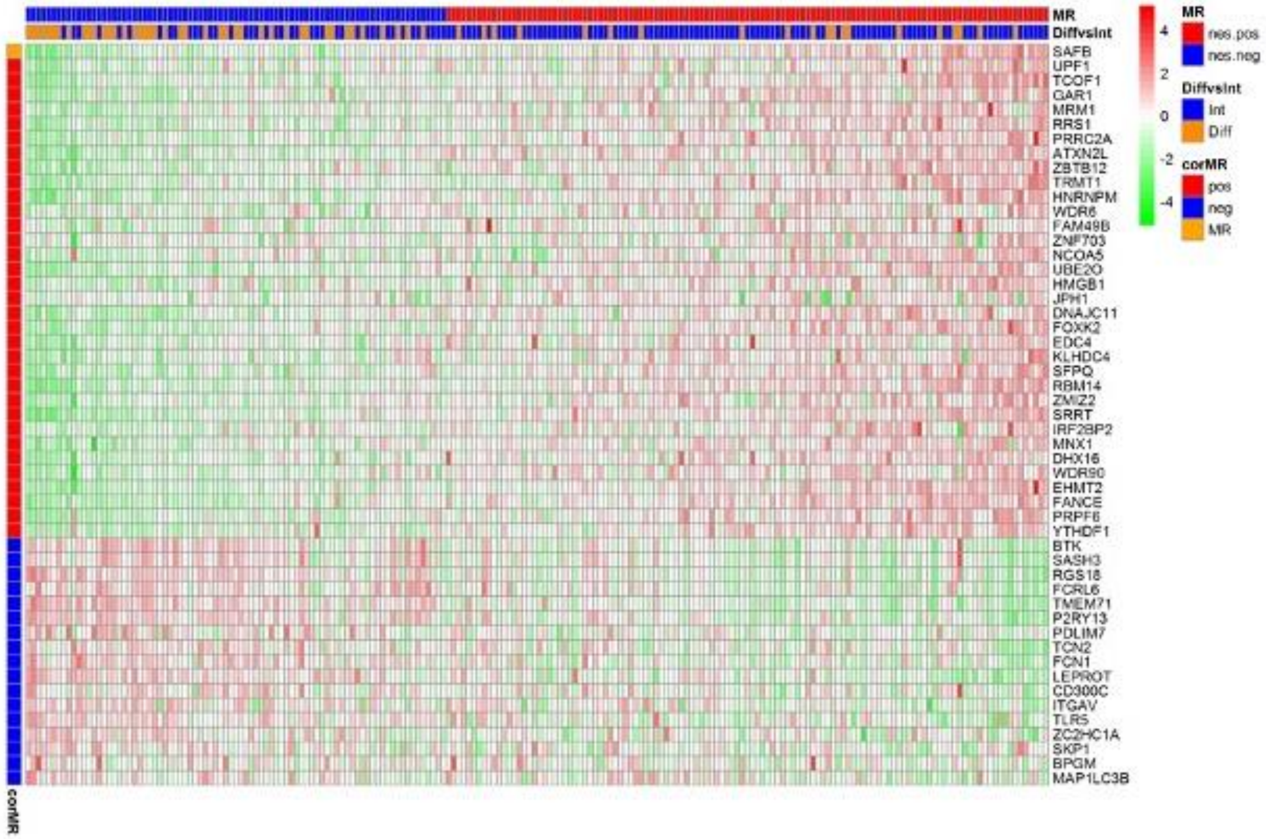

Figure S21. Heatmap of SAFB regulon associated with a better prognosis and with high activity in Intestinal tumors. Expression in red-green color scale. In legend, above the HM, Diffuse samples in orange while Intestinal samples in blue; in red samples with positive SAFB NES, in blue with negative SAFB NES. On the left of the HM, genes with positive correlation with the MR in red, genes with negative correlation in blue and the MR expression in orange.

Table S1. Summary of the GC clinical data

Characteristics		N = 377 patients
Age (years, median with range)		67 (30 - 90)
Gender	FEMALE	133 (35 %)
	MALE	244 (65 %)
Histological type	Diffuse	62 (16 %)
	Signet	12 (3 %)
	Stomach NOS	139 (37 %)
	Intestinal Mucinous	17 (5 %)
	Intestinal Papillary	7 (2 %)
	Intestinal Tubular	71 (19 %)
	Intestinal NOS	69 (18 %)
Anatomic	Antrum Distal	136 (38 %)
	Cardia Proximal	50 (14 %)
	Fundus Body	139 (39 %)
	Gastroesophageal Junction	36 (10 %)
	Missing	16 (4.24 %)
Anatomic JGCA	Distal	136 (42 %)
	Proximal	189 (58 %)
	Missing	52 (13.79 %)
Pathologic stage	I	52 (15 % %)
	II	110 (31 %)
	III	154 (44 %)
	IV	38 (11 %)
	Missing	23 (6.10 %)
Pathologic T	T1	20 (5 % %)
	T2	83 (23 %)
	T3	157 (43 %)
	T4	108 (29 %)
	Missing	9 (2.39 %)
Pathologic N	N0	114 (32 % %)
	N1	97 (27 %)
	N2	76 (21 %)
	N3	73 (20 %)
	Missing	17 (4.51 %)
Pathologic M	M0	334 (93 %)
	M1	25 (7 %)
	Missing	18 (4.77 %)
Microsatellite	MSS	248 (66 %)
	MSI.L	54 (14 %)
	MSI.H	74 (20 %)
	Missing	1 (0.27 %)
Primary therapy outcome success	CR	213 (69 %)
	PR	6 (2 %)
	SD	28 (9 %)
	PD	63 (20 %)
	Missing	67 (17.77 %)

NOS: Not Otherwise Specified; JCGA: Japanese Gastric Cancer Association; MSS: Microsatellite Stable; MSI.L: Microsatellite Instable Low; MSI.H: Microsatellite Instable High; CR: Complete Response; PR: Partial Response; SD: Stable Disease; PD: Progressive Disease.
